# Supplementary material for: Ambient particulate matter pollution and adult hospital admissions for pneumonia in urban China: A national time series analysis for 2014 through 2017
Source: PLoS Med. 2019 Dec 31;16(12):e1003010. doi: 10.1371/journal.pmed.1003010 (PMC6938337; doi:10.1371/journal.pmed.1003010)
Supplement: S1 Table — (DOCX) [file pmed.1003010.s001.docx]

**S1 Table:** Summary statistics on city-level characteristics in 184 Chinese cities.

| City | Number of city's residents (thousand)^*^ | Number of people enrolled in the UEBMI in 2017 (thousand) | Coverage rate of the UEBMI (%) | Annual average PM_2.5_ concentrations (μg/m^3^) | Annual average PM_10_ concentrations (μg/m^3^) | Annual average temperature (°C) | Annual average relative humidity (%) | Years of the study period (year) | Annual average daily pneumonia hospitalizations |
| --- | --- | --- | --- | --- | --- | --- | --- | --- | --- |
| An'shan | 3645.9 | 1169.735 | 32.08 | 64 | 64 | 11 | 50 | 4 | 17 |
| Anyang | 5172.8 | 639.594 | 12.36 | 91 | 91 | 15 | 59 | 4 | 13 |
| Baicheng | 2033.1 | 238.969 | 11.75 | 59 | 58 | 4 | 56 | 3 | 4 |
| Baise | 3466.8 | 352.491 | 10.17 | 40 | 40 | 23 | 78 | 3 | 11 |
| Baiyin | 1708.8 | 267.624 | 15.66 | 37 | 37 | 12 | 55 | 3 | 8 |
| Baoji | 3716.7 | 578.582 | 15.57 | 61 | 61 | 13 | 66 | 4 | 36 |
| Baoshan | 2506 | 162.638 | 6.49 | 28 | 28 | 17 | 67 | 3 | 6 |
| Baotou | 2650.3 | 881.293 | 33.25 | 49 | 49 | 8 | 55 | 4 | 11 |
| Bayannur | 1669.9 | 258.573 | 15.48 | 41 | 41 | 7 | 45 | 3 | 4 |
| Bazhong | 3283.7 | 215.918 | 6.58 | 35 | 35 | 18 | 75 | 3 | 8 |
| Beihai | 1539.3 | 248.703 | 16.16 | 26 | 26 | 24 | 83 | 4 | 3 |
| Bengbu | 3164 | 518.759 | 16.40 | 61 | 61 | 16 | 77 | 3 | 5 |
| Benxi | 1709.5 | 783.511 | 45.83 | 49 | 49 | 9 | 60 | 4 | 16 |
| Binzhou | 3748.5 | 643.217 | 17.16 | 77 | 77 | 14 | 61 | 4 | 3 |
| Bozhou | 4851 | 293.575 | 6.05 | 60 | 60 | 17 | 71 | 3 | 3 |
| Changde | 5717.2 | 515.205 | 9.01 | 56 | 56 | 18 | 78 | 4 | 25 |
| Changsha | 7044.1 | 1226.816 | 17.42 | 62 | 62 | 18 | 78 | 4 | 58 |
| Changzhi | 3334.6 | 649.839 | 19.49 | 64 | 64 | 11 | 58 | 4 | 5 |
| Changzhou | 4592 | 2082.949 | 45.36 | 57 | 57 | 17 | 73 | 4 | 26 |
| Chaoyang | 3044.6 | 496.376 | 16.30 | 42 | 42 | 11 | 52 | 3 | 15 |
| Chengde | 3473.2 | 543.004 | 15.63 | 38 | 38 | 10 | 56 | 4 | 7 |
| Chengdu | 14047.6 | 8084.426 | 57.55 | 64 | 64 | 16 | 82 | 4 | 376 |
| Chenzhou | 4581.8 | 483.704 | 10.56 | 41 | 41 | 19 | 79 | 3 | 19 |
| Chifeng | 4341.2 | 652.651 | 15.03 | 39 | 39 | 8 | 48 | 4 | 6 |
| Chizhou | 1403 | 156.549 | 11.16 | 54 | 54 | 18 | 75 | 3 | 3 |
| Chongqing | 28846.2 | 7033.574 | 24.38 | 56 | 56 | 19 | 77 | 4 | 314 |
| Chongzuo | 1194.3 | 186.098 | 15.58 | 33 | 33 | 23 | 77 | 3 | 3 |
| Chuzhou | 3938 | 485.798 | 12.34 | 58 | 58 | 16 | 80 | 3 | 10 |
| Dalian | 6690.4 | 4739.327 | 70.84 | 43 | 43 | 13 | 61 | 4 | 73 |
| Dandong | 2444.7 | 842.188 | 34.45 | 39 | 39 | 11 | 69 | 4 | 25 |
| Datong | 3318.1 | 694.392 | 20.93 | 38 | 38 | 8 | 56 | 4 | 22 |
| Daxinganling | 511.6 | 114.424 | 22.37 | 21 | 21 | 2 | 67 | 3 | 9 |
| Dazhou | 5468.1 | 430.925 | 7.88 | 55 | 55 | 18 | 77 | 3 | 14 |
| Dezhou | 5568.2 | 699.408 | 12.56 | 89 | 89 | 14 | 67 | 4 | 9 |
| Dongying | 2035.3 | 534.658 | 26.27 | 68 | 68 | 15 | 61 | 4 | 3 |
| Erdos | 1940.7 | 438.985 | 22.62 | 26 | 26 | 8 | 47 | 4 | 4 |
| Fangchenggang | 866.9 | 131.863 | 15.21 | 28 | 28 | 24 | 81 | 3 | 4 |
| Fushun | 2138.1 | 920.459 | 43.05 | 50 | 50 | 7 | 66 | 4 | 31 |
| Guangyuan | 2484.1 | 392.252 | 15.79 | 24 | 24 | 16 | 69 | 3 | 15 |
| Guangzhou | 12700.8 | 12970.73 | 102.13 | 39 | 39 | 22 | 80 | 4 | 83 |
| Guigang | 4118.8 | 271.472 | 6.59 | 40 | 40 | 23 | 82 | 3 | 2 |
| Guyuan | 1228.2 | 115.925 | 9.44 | 37 | 37 | 8 | 55 | 3 | 1 |
| Haikou | 2046.1 | 748.422 | 36.58 | 23 | 23 | 24 | 82 | 4 | 7 |
| Handan | 9174.6 | 1119.457 | 12.20 | 92 | 92 | 15 | 58 | 4 | 8 |
| Hangzhou | 8700.4 | 6671.225 | 76.68 | 53 | 53 | 18 | 74 | 4 | 50 |
| Hefei | 5702 | 2263.288 | 39.69 | 49 | 49 | 20 | 75 | 4 | 27 |
| Hegang | 1058.7 | 128.822 | 12.17 | 37 | 37 | 6 | 66 | 3 | 3 |
| Heihe | 1633.9 | 114.306 | 7.00 | 25 | 25 | 3 | 63 | 3 | 3 |
| Hengshui | 4340.8 | 382.178 | 8.80 | 102 | 102 | 11 | 58 | 4 | 1 |
| Hengyang | 7141.5 | 850.294 | 11.91 | 51 | 52 | 19 | 75 | 3 | 12 |
| Hetian | 2014.3 | 181.223 | 9.00 | 112 | 112 | 15 | 36 | 3 | 6 |
| Heze | 8287.8 | 705.687 | 8.51 | 86 | 86 | 15 | 69 | 4 | 9 |
| Hezhou | 1954.1 | 178.734 | 9.15 | 37 | 37 | 22 | 79 | 3 | 5 |
| Hinggan League | 1613.3 | 229.829 | 14.25 | 29 | 29 | 7 | 48 | 3 | 4 |
| Hohhot | 2866.6 | 755.669 | 26.36 | 43 | 43 | 7 | 47 | 4 | 4 |
| Huai'an | 4799.9 | 924.739 | 19.27 | 55 | 55 | 16 | 77 | 4 | 14 |
| Huaibei | 2114 | 438.918 | 20.76 | 59 | 59 | 16 | 67 | 3 | 12 |
| Huaihua | 4741.9 | 402.953 | 8.50 | 44 | 44 | 18 | 80 | 3 | 24 |
| Huainan | 2334 | 530.365 | 22.72 | 57 | 57 | 17 | 77 | 3 | 37 |
| Huangshan | 1359 | 215.696 | 15.87 | 28 | 28 | 10 | 81 | 3 | 7 |
| Huludao | 2623.5 | 660.431 | 25.17 | 51 | 51 | 11 | 53 | 4 | 23 |
| Hulunbeier | 2549.3 | 708.438 | 27.79 | 28 | 28 | 0 | 62 | 3 | 8 |
| Jiaxing | 4501.7 | 2526.202 | 56.12 | 49 | 49 | 17 | 82 | 4 | 29 |
| Jiayuguan | 231.9 | 99.73 | 43.01 | 28 | 28 | 9 | 47 | 4 | 4 |
| Jilin | 4414.7 | 938.347 | 21.26 | 54 | 54 | 5 | 65 | 4 | 16 |
| Jinan | 6814 | 3198.532 | 46.94 | 79 | 79 | 15 | 56 | 4 | 31 |
| Jincheng | 2279.2 | 524.833 | 23.03 | 62 | 62 | 11 | 59 | 3 | 4 |
| Jinhua | 5361.6 | 1639.551 | 30.58 | 52 | 52 | 19 | 74 | 4 | 13 |
| Jining | 8081.9 | 1313.794 | 16.26 | 74 | 74 | 15 | 70 | 4 | 31 |
| Jinzhong | 3249.4 | 644.623 | 19.84 | 60 | 60 | 12 | 58 | 3 | 7 |
| Jinzhou | 3126.4 | 838.432 | 26.82 | 56 | 56 | 11 | 53 | 4 | 18 |
| Jixi | 1862.1 | 284.222 | 15.26 | 33 | 34 | 7 | 64 | 3 | 2 |
| Karamay | 391 | 77.782 | 19.89 | 30 | 30 | 11 | 47 | 4 | 1 |
| Kashi | 3979.3 | 375.495 | 9.44 | 143 | 139 | 11 | 46 | 3 | 9 |
| Kiamusze | 2552.1 | 437.396 | 17.14 | 26 | 26 | 8 | 71 | 3 | 7 |
| Kunming | 6432 | 1715.482 | 26.67 | 29 | 29 | 16 | 70 | 4 | 47 |
| Laibin | 2099.7 | 194.652 | 9.27 | 43 | 43 | 22 | 79 | 3 | 2 |
| Laiwu | 1298.5 | 238.67 | 18.38 | 81 | 81 | 14 | 62 | 4 | 6 |
| Langfang | 4358.8 | 1140.862 | 26.17 | 72 | 72 | 11 | 61 | 4 | 4 |
| Lanzhou | 3616.2 | 936.616 | 25.90 | 52 | 52 | 10 | 58 | 4 | 16 |
| Lianyungang | 4393.9 | 1070.188 | 24.36 | 43 | 43 | 17 | 73 | 4 | 6 |
| Liaocheng | 5789.9 | 695.739 | 12.02 | 89 | 89 | 14 | 65 | 4 | 20 |
| Liaoyang | 1858.8 | 620.288 | 33.37 | 51 | 51 | 9 | 61 | 3 | 26 |
| Liaoyuan | 1176.6 | 209.678 | 17.82 | 48 | 48 | 7 | 66 | 3 | 4 |
| Lijiang | 1245 | 128.848 | 10.35 | 15 | 15 | 14 | 61 | 3 | 4 |
| Linfen | 4316.6 | 635.71 | 14.73 | 89 | 89 | 14 | 54 | 4 | 7 |
| Lishui | 2117 | 448.88 | 21.20 | 36 | 36 | 19 | 74 | 3 | 9 |
| Liu'an | 5612 | 379.776 | 6.77 | 50 | 50 | 17 | 76 | 3 | 8 |
| Liuzhou | 3758.7 | 1106.096 | 29.43 | 45 | 45 | 22 | 72 | 4 | 16 |
| Longnan | 2567.7 | 131.365 | 5.12 | 26 | 26 | 14 | 67 | 3 | 1 |
| Loudi | 3785.6 | 501.615 | 13.25 | 47 | 47 | 18 | 80 | 3 | 8 |
| Lvliang | 3727.1 | 361.492 | 9.70 | 62 | 62 | 11 | 60 | 3 | 2 |
| Ma'anshan | 1366 | 516.606 | 37.82 | 56 | 56 | 17 | 77 | 4 | 10 |
| Maoming | 5817.8 | 620.851 | 10.67 | 38 | 38 | 19 | 85 | 4 | 18 |
| Mianyang | 4613.9 | 747.878 | 16.21 | 68 | 68 | 13 | 71 | 4 | 58 |
| Mudanjiang | 2798.7 | 23.65 | 0.85 | 35 | 35 | 5 | 65 | 4 | 1 |
| Nanchang | 5042.6 | 1235.277 | 24.50 | 43 | 43 | 19 | 75 | 4 | 19 |
| Nanchong | 6278.6 | 930.867 | 14.83 | 61 | 61 | 18 | 79 | 4 | 13 |
| Nanjing | 8004.7 | 4714.89 | 58.90 | 55 | 55 | 17 | 73 | 4 | 81 |
| Nanning | 6661.6 | 1930.216 | 28.98 | 37 | 37 | 21 | 81 | 4 | 14 |
| Nantong | 7282.8 | 2258.199 | 31.01 | 51 | 51 | 16 | 78 | 4 | 72 |
| Ningbo | 7605.7 | 7823.121 | 102.86 | 41 | 41 | 18 | 76 | 4 | 35 |
| Panjin | 1392.5 | 345.817 | 24.83 | 46 | 47 | 10 | 60 | 4 | 15 |
| Pu'er | 2543 | 233.122 | 9.17 | 26 | 26 | 20 | 76 | 3 | 10 |
| Qingyang | 2211.2 | 165.467 | 7.48 | 36 | 36 | 11 | 59 | 3 | 3 |
| Qingyuan | 3698.4 | 1631.227 | 44.11 | 38 | 38 | 22 | 77 | 4 | 15 |
| Qinhuangdao | 2987.6 | 525.617 | 17.59 | 49 | 49 | 11 | 68 | 4 | 5 |
| Qinzhou | 3079.7 | 242.058 | 7.86 | 34 | 34 | 24 | 81 | 3 | 9 |
| Qiqihar | 5367 | 614.791 | 11.46 | 33 | 33 | 9 | 62 | 4 | 2 |
| Qujing | 5855 | 518.546 | 8.86 | 31 | 31 | 16 | 70 | 4 | 23 |
| Quzhou | 2122.7 | 805.094 | 37.93 | 45 | 45 | 19 | 79 | 4 | 12 |
| Sanya | 685.4 | 470.121 | 68.59 | 18 | 18 | 23 | 92 | 4 | 8 |
| Shangrao | 6579.7 | 468.295 | 7.12 | 43 | 43 | 19 | 78 | 3 | 11 |
| Shantou | 5391 | 583.743 | 10.83 | 33 | 33 | 23 | 77 | 4 | 9 |
| Shaoxing | 4912.2 | 1871.129 | 38.09 | 52 | 52 | 18 | 73 | 4 | 15 |
| Shaoyang | 7071.8 | 521.997 | 7.38 | 56 | 56 | 18 | 81 | 3 | 30 |
| Shenyang | 8106.2 | 3397.203 | 41.91 | 61 | 61 | 9 | 60 | 4 | 106 |
| Shiyan | 3340.8 | 607.559 | 18.19 | 50 | 50 | 16 | 70 | 3 | 7 |
| Shizuishan | 725.5 | 216.019 | 29.78 | 45 | 45 | 11 | 46 | 4 | 6 |
| Shuozhou | 1714.9 | 226.59 | 13.21 | 53 | 53 | 8 | 52 | 3 | 6 |
| Siping | 3386.3 | 194.77 | 5.75 | 52 | 52 | 8 | 63 | 3 | 4 |
| Suzhou | 10466 | 7157.478 | 68.39 | 53 | 53 | 16 | 78 | 4 | 122 |
| Suzhou | 5353 | 333.895 | 6.24 | 66 | 53 | 17 | 72 | 4 | 122 |
| Taian | 5494.2 | 1085.073 | 19.75 | 67 | 67 | 7 | 65 | 4 | 21 |
| Taiyuan | 4201.6 | 2481.991 | 59.07 | 71 | 71 | 6 | 48 | 4 | 29 |
| Taizhou | 4618.6 | 1540.62 | 33.36 | 69 | 69 | 17 | 78 | 4 | 19 |
| Taizhou | 5968.8 | 2617.891 | 43.86 | 39 | 69 | 19 | 78 | 4 | 19 |
| Tangshan | 7577.3 | 1647.621 | 21.74 | 84 | 84 | 12 | 63 | 4 | 12 |
| Tianjin | 12938.2 | 5684.847 | 43.94 | 71 | 71 | 14 | 57 | 4 | 72 |
| Tieling | 2717.7 | 648.938 | 23.88 | 52 | 52 | 8 | 61 | 3 | 12 |
| Tonghua | 2325.2 | 333.637 | 14.35 | 43 | 43 | 7 | 67 | 3 | 2 |
| Tongliao | 3139.2 | 362.058 | 11.53 | 43 | 43 | 8 | 52 | 3 | 3 |
| Tongling | 724 | 347.393 | 47.98 | 53 | 53 | 18 | 81 | 3 | 9 |
| Turpan | 622.7 | 115.743 | 18.59 | 68 | 68 | 16 | 34 | 3 | 5 |
| Ulanqab | 2143.6 | 288.938 | 13.48 | 36 | 36 | 6 | 47 | 3 | 6 |
| Weifang | 9086.2 | 2003.457 | 22.05 | 68 | 68 | 14 | 62 | 4 | 31 |
| Weihai | 2804.8 | 1097.634 | 39.13 | 35 | 35 | 14 | 62 | 4 | 11 |
| Wenzhou | 9122.1 | 2416.055 | 26.49 | 42 | 42 | 18 | 81 | 4 | 10 |
| Wuhai | 532.9 | 216.1 | 40.55 | 48 | 48 | 11 | 44 | 3 | 6 |
| Wuhan | 9785.4 | 4789.449 | 48.94 | 67 | 67 | 16 | 80 | 4 | 169 |
| Wuhu | 2263 | 828.802 | 36.62 | 56 | 56 | 17 | 78 | 4 | 36 |
| Wuwei | 1815.1 | 153.369 | 8.45 | 41 | 41 | 10 | 44 | 3 | 2 |
| Wuxi | 6372.6 | 3983.419 | 62.51 | 56 | 56 | 17 | 74 | 4 | 53 |
| Wuzhong | 1273.8 | 169.305 | 13.29 | 47 | 47 | 11 | 47 | 3 | 3 |
| Xiangtan | 2748.6 | 497.425 | 18.10 | 59 | 59 | 17 | 81 | 4 | 15 |
| Xiangyang | 5500.3 | 842.67 | 15.32 | 68 | 68 | 17 | 71 | 3 | 37 |
| Xiaogan | 4814.5 | 467.471 | 9.71 | 46 | 46 | 18 | 76 | 3 | 9 |
| Xilin Gol League | 1028 | 261.29 | 25.42 | 16 | 16 | 5 | 53 | 3 | 2 |
| Xingtai | 7104.1 | 731.288 | 10.29 | 88 | 88 | 17 | 59 | 4 | 6 |
| Xining | 2208.7 | 395.352 | 17.90 | 44 | 44 | 7 | 58 | 4 | 6 |
| Xuancheng | 3067.5 | 417.393 | 13.61 | 49 | 49 | 18 | 79 | 3 | 9 |
| Ya'an | 2533 | 438.373 | 17.31 | 41 | 41 | 16 | 80 | 3 | 7 |
| Yancheng | 1507.2 | 297.842 | 19.76 | 48 | 48 | 15 | 79 | 4 | 27 |
| Yangquan | 7260.2 | 1400.917 | 19.30 | 63 | 63 | 15 | 57 | 4 | 3 |
| Yangzhou | 1368.5 | 280.312 | 20.48 | 57 | 57 | 16 | 73 | 4 | 43 |
| Yantai | 4459.8 | 1427.845 | 32.02 | 43 | 43 | 13 | 64 | 4 | 26 |
| Yibin | 6968.2 | 3138.85 | 45.05 | 58 | 58 | 19 | 77 | 4 | 15 |
| Yichang | 4059.7 | 830.897 | 20.47 | 71 | 71 | 17 | 77 | 4 | 15 |
| Yichun | 4059.7 | 1112.95 | 27.41 | 23 | 23 | 2 | 68 | 3 | 6 |
| Yinchuan | 1148.1 | 346.149 | 30.15 | 51 | 51 | 9 | 49 | 4 | 11 |
| Yingkou | 1993.1 | 782.664 | 39.27 | 44 | 44 | 11 | 63 | 4 | 16 |
| Yingtan | 2428.5 | 875.818 | 36.06 | 44 | 56 | 15 | 82 | 3 | 8 |
| Yiyang | 1124.9 | 121.118 | 10.77 | 46 | 46 | 17 | 78 | 3 | 12 |
| Yizhou | 4313.1 | 384.624 | 8.92 | 59 | 59 | 11 | 48 | 3 | 6 |
| Yongzhou | 5180.2 | 380.187 | 7.34 | 48 | 48 | 19 | 81 | 3 | 3 |
| Yueyang | 5477.9 | 605.061 | 11.05 | 52 | 52 | 18 | 79 | 4 | 21 |
| Yulin | 5487.4 | 400.719 | 7.30 | 38 | 38 | 22 | 81 | 3 | 7 |
| Yuncheng | 5134.8 | 596.231 | 11.61 | 67 | 67 | 15 | 60 | 3 | 6 |
| Yunfu | 2360.1 | 266.894 | 11.31 | 36 | 36 | 24 | 83 | 4 | 1 |
| Yuxi | 2304 | 283.435 | 12.30 | 26 | 26 | 17 | 71 | 4 | 13 |
| Zaozhuang | 3729.3 | 508.935 | 13.65 | 79 | 79 | 15 | 64 | 4 | 10 |
| Zhangjiajie | 1476.5 | 131.962 | 8.94 | 48 | 48 | 17 | 81 | 4 | 8 |
| Zhangjiakou | 4345.5 | 846.879 | 19.49 | 33 | 33 | 12 | 50 | 4 | 12 |
| Zhangye | 1199.5 | 99.579 | 8.30 | 35 | 51 | -2 | 49 | 3 | 4 |
| Zhaoqing | 3918.1 | 827.695 | 21.12 | 80 | 36 | 16 | 70 | 3 | 19 |
| Zhaotong | 5213 | 236.61 | 4.54 | 32 | 32 | 13 | 74 | 3 | 13 |
| Zhenjiang | 3113.3 | 1012.385 | 32.52 | 58 | 58 | 17 | 72 | 4 | 6 |
| Zhongshan | 3120.9 | 3035.102 | 97.25 | 34 | 34 | 23 | 78 | 4 | 7 |
| Zhongwei | 1080.8 | 133.203 | 12.32 | 43 | 43 | 10 | 50 | 3 | 3 |
| Zhoushan | 1121.3 | 424.164 | 37.83 | 28 | 28 | 17 | 81 | 4 | 6 |
| Zhuzhou | 3855.6 | 665.169 | 17.25 | 58 | 58 | 18 | 79 | 4 | 22 |
| Zibo | 4530.6 | 1521.334 | 33.58 | 80 | 80 | 14 | 62 | 4 | 34 |
| Zigong | 2678.9 | 467.664 | 17.46 | 73 | 73 | 17 | 82 | 4 | 29 |
| Ziyang | 3665.1 | 218.918 | 5.97 | 44 | 44 | 18 | 80 | 3 | 6 |

PM_2.5_, particulate matter ≤2.5 μm in aerodynamic diameter; PM_10_, particulate matter ≤10 μm in aerodynamic diameter.

^*^ The number of city residents is according to the 2010 census.
