# Supplementary material for: Ambient particulate matter pollution and adult hospital admissions for pneumonia in urban China: A national time series analysis for 2014 through 2017
Source: PLoS Med. 2019 Dec 31;16(12):e1003010. doi: 10.1371/journal.pmed.1003010 (PMC6938337; doi:10.1371/journal.pmed.1003010)
Supplement: S2 Table — (DOCX) [file pmed.1003010.s002.docx]

**S2 Table.** City-specific percentage increase with 95% confidence interval^*^ in daily hospital admissions for pneumonia associated with a 10 μg/m^3^ increase in PM_2.5_ and PM_10_ concentrations (lag 0–2) in 184 Chinese cities, 2014–2017.

| **City** | **PM_2.5_** | | |  | **PM_10_** |  |
| --- | --- | --- | --- | --- | --- | --- |
|  | Percentage increase (95% confidence interval) | | | *P* value | Percentage increase (95% confidence interval) | *P* value |
| **Unadjusted analyses** | | |  |  |  |  |
| An'shan | 0.66 (-0.71 to 2.04) | | | 0.347 | 0.62 (-0.45 to 1.7) | 0.259 |
| Anyang | -0.15 (-0.78 to 0.49) | | | 0.646 | 0.08 (-0.37 to 0.53) | 0.728 |
| Baicheng | 4.53 (0.49 to 8.73) | | | 0.028 | 2.36 (-0.78 to 5.6) | 0.143 |
| Baise | -1.06 (-3.23 to 1.16) | | | 0.346 | -0.61 (-2.33 to 1.15) | 0.496 |
| Baiyin | -2.86 (-5.38 to -0.27) | | | 0.031 | -0.89 (-1.69 to -0.09) | 0.031 |
| Baoji | 0.25 (-0.33 to 0.84) | | | 0.397 | 0.2 (-0.22 to 0.61) | 0.353 |
| Baoshan | -0.14 (-4.27 to 4.16) | | | 0.946 | 1.46 (-0.9 to 3.88) | 0.228 |
| Baotou | -0.27 (-1.22 to 0.7) | | | 0.587 | 0.07 (-0.39 to 0.53) | 0.774 |
| Bayannur | -0.37 (-2.03 to 1.32) | | | 0.665 | -0.27 (-0.84 to 0.31) | 0.365 |
| Bazhong | -1.26 (-3.84 to 1.4) | | | 0.35 | -0.28 (-2.16 to 1.63) | 0.772 |
| Beihai | 2.54 (-1.74 to 7.01) | | | 0.249 | 1.21 (-2.21 to 4.74) | 0.495 |
| Bengbu | 1.23 (-0.85 to 3.35) | | | 0.248 | 0.1 (-1.05 to 1.26) | 0.863 |
| Benxi | -1.07 (-2.09 to -0.03) | | | 0.043 | -1.16 (-1.97 to -0.35) | 0.005 |
| Binzhou | -0.56 (-1.58 to 0.47) | | | 0.283 | -0.29 (-1 to 0.43) | 0.428 |
| Bozhou | 0.75 (-1.08 to 2.62) | | | 0.424 | 0.32 (-1 to 1.65) | 0.638 |
| Changde | 1.01 (-0.17 to 2.2) | | | 0.095 | 0.98 (0.06 to 1.91) | 0.037 |
| Changsha | -1.16 (-2.46 to 0.16) | | | 0.085 | -0.8 (-1.87 to 0.27) | 0.143 |
| Changzhi | 0.87 (-0.66 to 2.44) | | | 0.266 | 0.35 (-0.76 to 1.47) | 0.542 |
| Changzhou | 0.53 (-0.22 to 1.29) | | | 0.169 | 0.43 (-0.07 to 0.93) | 0.093 |
| Chaoyang | -0.39 (-1.47 to 0.71) | | | 0.487 | -0.23 (-0.98 to 0.52) | 0.547 |
| Chengde | -1.28 (-3.25 to 0.74) | | | 0.213 | -0.64 (-1.57 to 0.3) | 0.179 |
| Chengdu | -0.19 (-1.5 to 1.14) | | | 0.778 | 0.29 (-0.6 to 1.18) | 0.529 |
| Chenzhou | 0.04 (-1.66 to 1.77) | | | 0.965 | 0.2 (-0.9 to 1.32) | 0.723 |
| Chifeng | 0.35 (-0.99 to 1.71) | | | 0.609 | 0.62 (-0.11 to 1.36) | 0.094 |
| Chizhou | 1.74 (-3.02 to 6.73) | | | 0.48 | 1.18 (-1.92 to 4.37) | 0.461 |
| Chongqing | -0.18 (-1.04 to 0.69) | | | 0.682 | 0.02 (-0.59 to 0.64) | 0.941 |
| Chongzuo | 2.51 (-2.01 to 7.25) | | | 0.282 | 1.79 (-1.71 to 5.42) | 0.321 |
| Chuzhou | -0.2 (-1.67 to 1.29) | | | 0.794 | -0.01 (-1.06 to 1.06) | 0.992 |
| Dalian | -0.24 (-1.51 to 1.04) | | | 0.712 | -0.63 (-1.58 to 0.32) | 0.191 |
| Dandong | 0.48 (-1.46 to 2.46) | | | 0.628 | 0.29 (-1.17 to 1.77) | 0.702 |
| Datong | -1.2 (-2.77 to 0.4) | | | 0.141 | -0.34 (-1.23 to 0.56) | 0.461 |
| Daxinganling | -2.19 (-9.97 to 6.27) | | | 0.602 | -4.42 (-10.03 to 1.54) | 0.143 |
| Dazhou | 1.06 (-0.18 to 2.31) | | | 0.094 | 0.55 (-0.34 to 1.45) | 0.229 |
| Dezhou | -0.09 (-0.62 to 0.44) | | | 0.731 | -0.17 (-0.53 to 0.2) | 0.37 |
| Dongying | 0.01 (-1.05 to 1.08) | | | 0.988 | -0.01 (-0.72 to 0.71) | 0.98 |
| Erdos | 1.29 (-0.79 to 3.41) | | | 0.226 | -0.06 (-0.75 to 0.63) | 0.854 |
| Fangchenggang | 1.68 (-3.81 to 7.49) | | | 0.556 | 2.05 (-2.18 to 6.47) | 0.348 |
| Fushun | -1.56 (-2.5 to -0.62) | | | 0.001 | -0.88 (-1.53 to -0.22) | 0.009 |
| Guangyuan | -4.23 (-6.49 to -1.91) | | | <0.001 | -1.18 (-2.38 to 0.02) | 0.055 |
| Guangzhou | 0.1 (-1.55 to 1.78) | | | 0.904 | 0.31 (-0.84 to 1.47) | 0.602 |
| Guigang | 1.14 (-3.21 to 5.68) | | | 0.614 | 1.4 (-1.53 to 4.43) | 0.353 |
| Guyuan | -4.45 (-10.9 to 2.47) | | | 0.203 | -1.6 (-3.99 to 0.84) | 0.197 |
| Haikou | 1.75 (-2.97 to 6.7) | | | 0.474 | 1.97 (-1.42 to 5.48) | 0.258 |
| Handan | 0.11 (-0.45 to 0.68) | | | 0.7 | -0.32 (-0.73 to 0.08) | 0.121 |
| Hangzhou | -0.52 (-1.37 to 0.35) | | | 0.239 | -0.28 (-0.86 to 0.3) | 0.338 |
| Hefei | 5.95 (3.15 to 8.83) | | | <0.001 | 3.46 (1.48 to 5.47) | 0.001 |
| Hegang | -0.48 (-4.32 to 3.51) | | | 0.81 | 0.01 (-2.54 to 2.63) | 0.992 |
| Heihe | -10.73 (-23.83 to 4.63) | | | 0.161 | -0.58 (-7.14 to 6.44) | 0.866 |
| Hengshui | 0.46 (-2.32 to 3.32) | | | 0.747 | 0.63 (-1.3 to 2.59) | 0.527 |
| Hengyang | 0.78 (-0.78 to 2.36) | | | 0.328 | 0.43 (-0.64 to 1.52) | 0.429 |
| Hetian | -0.7 (-2.34 to 0.98) | | | 0.415 | -0.27 (-1.05 to 0.52) | 0.502 |
| Heze | 0.45 (-0.16 to 1.06) | | | 0.145 | 0.26 (-0.17 to 0.7) | 0.237 |
| Hezhou | 0.46 (-2.61 to 3.64) | | | 0.771 | 0.72 (-1.38 to 2.87) | 0.506 |
| Hinggan League | -0.67 (-3.22 to 1.94) | | | 0.612 | -0.59 (-1.95 to 0.78) | 0.395 |
| Hohhot | -0.27 (-1.87 to 1.36) | | | 0.747 | -0.24 (-0.95 to 0.48) | 0.511 |
| Huai'an | -0.25 (-1.18 to 0.7) | | | 0.609 | -0.03 (-0.65 to 0.59) | 0.924 |
| Huaibei | -1.55 (-2.8 to -0.28) | | | 0.017 | -0.95 (-1.79 to -0.1) | 0.029 |
| Huaihua | 1.35 (-0.21 to 2.93) | | | 0.09 | 1.12 (0.21 to 2.05) | 0.016 |
| Huainan | 0.89 (-1.09 to 2.9) | | | 0.382 | 0.51 (-0.59 to 1.62) | 0.366 |
| Huangshan | -0.13 (-2.71 to 2.51) | | | 0.922 | 0.08 (-1.81 to 2.01) | 0.935 |
| Huludao | 0.38 (-0.76 to 1.52) | | | 0.517 | 0.07 (-0.68 to 0.83) | 0.858 |
| Hulunbeier | -0.32 (-3.09 to 2.52) | | | 0.821 | -0.5 (-1.74 to 0.76) | 0.434 |
| Jiaxing | 0.84 (-0.03 to 1.72) | | | 0.058 | 0.65 (0.04 to 1.26) | 0.036 |
| Jiayuguan | -2.13 (-4.91 to 0.74) | | | 0.145 | -1.46 (-2.54 to -0.37) | 0.009 |
| Jilin | -0.47 (-1.34 to 0.4) | | | 0.288 | 0.06 (-0.57 to 0.7) | 0.853 |
| Jinan | -0.02 (-0.57 to 0.54) | | | 0.951 | -0.1 (-0.46 to 0.26) | 0.58 |
| Jincheng | 0.02 (-1.18 to 1.23) | | | 0.98 | -0.27 (-1.06 to 0.53) | 0.502 |
| Jinhua | 0.34 (-1.1 to 1.79) | | | 0.647 | 0.02 (-1.06 to 1.1) | 0.978 |
| Jining | 0.27 (-0.34 to 0.87) | | | 0.388 | 0.15 (-0.25 to 0.55) | 0.462 |
| Jinzhong | 0.55 (-0.63 to 1.75) | | | 0.365 | 0.07 (-0.73 to 0.87) | 0.871 |
| Jinzhou | -0.84 (-1.57 to -0.11) | | | 0.025 | -0.43 (-1.02 to 0.17) | 0.159 |
| Jixi | 0.85 (-2.89 to 4.75) | | | 0.66 | 0.19 (-2.58 to 3.04) | 0.894 |
| Karamay | 81.12 (-65.04 to 838.3) | | | 0.481 | 16.36 (-29.69 to 92.59) | 0.557 |
| Kashi | 0.24 (-0.64 to 1.13) | | | 0.596 | 0.07 (-0.17 to 0.32) | 0.549 |
| Kiamusze | 4.37 (-5.31 to 15.04) | | | 0.39 | 3.6 (-2.07 to 9.6) | 0.22 |
| Kunming | 0 (-10.38 to 11.58) | | | 1 | 0 (-5.77 to 6.12) | 1 |
| Laibin | 3.99 (0.24 to 7.89) | | | 0.037 | 3.38 (0.45 to 6.4) | 0.024 |
| Laiwu | 0.81 (-0.14 to 1.76) | | | 0.094 | 0.74 (0.06 to 1.42) | 0.032 |
| Langfang | 0.68 (-0.49 to 1.87) | | | 0.256 | 0.59 (-0.29 to 1.49) | 0.191 |
| Lanzhou | -3.72 (-6.04 to -1.34) | | | 0.002 | -1.49 (-2.16 to -0.82) | <0.001 |
| Lianyungang | -0.33 (-2.82 to 2.23) | | | 0.799 | 0.24 (-1.19 to 1.69) | 0.746 |
| Liaocheng | -0.16 (-0.64 to 0.33) | | | 0.526 | -0.15 (-0.49 to 0.18) | 0.371 |
| Liaoyang | -1.18 (-1.98 to -0.37) | | | 0.004 | -1.06 (-1.7 to -0.41) | 0.001 |
| Liaoyuan | 0.14 (-2.22 to 2.55) | | | 0.909 | -0.92 (-2.75 to 0.94) | 0.33 |
| Lijiang | 7.14 (-6.35 to 22.58) | | | 0.315 | 2.84 (-4.77 to 11.06) | 0.475 |
| Linfen | 1.73 (0.47 to 3.01) | | | 0.008 | 1.25 (0.24 to 2.27) | 0.016 |
| Lishui | 0 (-8.53 to 9.32) | | | 1 | 0 (-6.49 to 6.93) | 1 |
| Liu'an | 0.3 (-1.48 to 2.11) | | | 0.742 | 0.56 (-0.62 to 1.77) | 0.353 |
| Liuzhou | 0.67 (-1.02 to 2.39) | | | 0.44 | 0.41 (-0.75 to 1.58) | 0.492 |
| Longnan | 72.05 (-24.29 to 290.96) | | | 0.198 | 64.19 (-14.23 to 214.31) | 0.137 |
| Loudi | 1.48 (-0.17 to 3.16) | | | 0.079 | 1.11 (0.02 to 2.22) | 0.046 |
| Lvliang | 3.69 (-0.32 to 7.86) | | | 0.072 | 2.06 (0.17 to 3.99) | 0.034 |
| Ma'anshan | -2.28 (-3.83 to -0.7) | | | 0.005 | -1.87 (-2.94 to -0.79) | 0.001 |
| Maoming | 0.62 (-8.25 to 10.33) | | | 0.896 | 0.92 (-5.69 to 7.99) | 0.791 |
| Mianyang | -2.86 (-10.88 to 5.89) | | | 0.512 | -2.47 (-9.24 to 4.81) | 0.497 |
| Mudanjiang | 1.76 (-2.79 to 6.51) | | | 0.455 | 1 (-2.11 to 4.22) | 0.532 |
| Nanchang | 4.12 (2.8 to 5.46) | | | <0.001 | 2.76 (1.91 to 3.63) | <0.001 |
| Nanchong | -1.05 (-2.2 to 0.1) | | | 0.075 | -0.87 (-1.64 to -0.09) | 0.028 |
| Nanjing | 1.14 (0.13 to 2.15) | | | 0.026 | 1.07 (0.41 to 1.72) | 0.001 |
| Nanning | 5.8 (2.35 to 9.36) | | | 0.001 | 4.26 (2.21 to 6.35) | <0.001 |
| Nantong | 0.33 (-0.34 to 1) | | | 0.34 | 0.21 (-0.25 to 0.66) | 0.373 |
| Ningbo | 0.16 (-0.72 to 1.04) | | | 0.724 | 0.32 (-0.29 to 0.93) | 0.302 |
| Panjin | -1.36 (-2.75 to 0.06) | | | 0.06 | -0.62 (-1.78 to 0.55) | 0.296 |
| Pu'er | -1.8 (-4.59 to 1.07) | | | 0.217 | 0.15 (-1.74 to 2.07) | 0.879 |
| Qingyang | 0.35 (-2.29 to 3.06) | | | 0.798 | -1.61 (-2.96 to -0.24) | 0.022 |
| Qingyuan | 1.07 (-0.15 to 2.31) | | | 0.086 | 1.2 (0.37 to 2.03) | 0.005 |
| Qinhuangdao | -0.15 (-1.21 to 0.93) | | | 0.788 | -0.18 (-0.89 to 0.55) | 0.634 |
| Qinzhou | 1.06 (-1.84 to 4.04) | | | 0.478 | 0.37 (-1.81 to 2.6) | 0.743 |
| Qiqihar | -1.09 (-7.6 to 5.87) | | | 0.752 | -3.79 (-8.34 to 1) | 0.12 |
| Qujing | 1.04 (-1.68 to 3.83) | | | 0.458 | 1.17 (-0.87 to 3.26) | 0.263 |
| Quzhou | 1.94 (0.75 to 3.15) | | | 0.001 | 0.37 (-0.49 to 1.24) | 0.398 |
| Sanya | 2.2 (-5.5 to 10.54) | | | 0.586 | 2.5 (-3.15 to 8.49) | 0.393 |
| Shangrao | 2.76 (0.26 to 5.32) | | | 0.031 | 2.81 (1.3 to 4.35) | <0.001 |
| Shantou | 1.16 (-1.17 to 3.55) | | | 0.331 | 0.73 (-0.89 to 2.37) | 0.378 |
| Shaoxing | 0.03 (-0.97 to 1.04) | | | 0.953 | 0.16 (-0.54 to 0.86) | 0.654 |
| Shaoyang | 0.18 (-1.01 to 1.39) | | | 0.764 | 0.4 (-0.47 to 1.29) | 0.367 |
| Shenyang | -0.08 (-0.67 to 0.52) | | | 0.792 | -0.07 (-0.56 to 0.41) | 0.767 |
| Shiyan | 0.98 (-0.62 to 2.61) | | | 0.23 | 0.63 (-0.52 to 1.8) | 0.286 |
| Shizuishan | 0.08 (-2.05 to 2.25) | | | 0.945 | -1.06 (-1.94 to -0.18) | 0.018 |
| Shuozhou | -0.16 (-1.91 to 1.62) | | | 0.857 | -0.35 (-1.51 to 0.83) | 0.56 |
| Siping | -0.44 (-1.87 to 1.02) | | | 0.555 | 0.66 (-0.41 to 1.74) | 0.229 |
| Suzhou | -0.17 (-0.7 to 0.35) | | | 0.514 | 0.13 (-0.27 to 0.53) | 0.532 |
| Suzhou | -0.17 (-0.7 to 0.35) | | | 0.514 | 0.13 (-0.27 to 0.53) | 0.532 |
| Taian | 0.63 (0.01 to 1.25) | | | 0.048 | 0.46 (0.02 to 0.91) | 0.039 |
| Taiyuan | 2.1 (-2.21 to 6.59) | | | 0.346 | -0.36 (-3.94 to 3.34) | 0.845 |
| Taizhou | -1.69 (-3.77 to 0.43) | | | 0.118 | -0.62 (-1.85 to 0.63) | 0.33 |
| Taizhou | -1.69 (-3.77 to 0.43) | | | 0.118 | -0.62 (-1.85 to 0.63) | 0.33 |
| Tangshan | 0.1 (-0.46 to 0.66) | | | 0.718 | 0.35 (-0.03 to 0.73) | 0.072 |
| Tianjin | -0.01 (-0.7 to 0.69) | | | 0.985 | 0.23 (-0.29 to 0.75) | 0.384 |
| Tieling | 0.23 (-0.67 to 1.14) | | | 0.619 | -0.11 (-0.84 to 0.62) | 0.761 |
| Tonghua | 0.65 (-2.15 to 3.53) | | | 0.651 | 0.45 (-1.37 to 2.3) | 0.632 |
| Tongliao | -0.4 (-2.02 to 1.25) | | | 0.633 | 0.09 (-0.78 to 0.97) | 0.834 |
| Tongling | 0.79 (-1.59 to 3.24) | | | 0.518 | 0.47 (-1.21 to 2.17) | 0.589 |
| Turpan | 1.67 (0.23 to 3.13) | | | 0.023 | 0.14 (-0.31 to 0.59) | 0.554 |
| Ulanqab | 0.56 (-1.08 to 2.22) | | | 0.506 | 0.44 (-0.23 to 1.12) | 0.199 |
| Weifang | 0.52 (-0.07 to 1.11) | | | 0.085 | 0.24 (-0.17 to 0.65) | 0.247 |
| Weihai | -0.09 (-1.27 to 1.1) | | | 0.877 | -0.61 (-1.4 to 0.18) | 0.131 |
| Wenzhou | -0.48 (-2.29 to 1.37) | | | 0.609 | 0.3 (-0.82 to 1.43) | 0.603 |
| Wuhai | -0.44 (-1.96 to 1.1) | | | 0.571 | -0.3 (-0.9 to 0.31) | 0.339 |
| Wuhan | 0.78 (-0.49 to 2.07) | | | 0.228 | 0.88 (0.04 to 1.72) | 0.04 |
| Wuhu | -1.62 (-3 to -0.22) | | | 0.024 | -1.1 (-2.09 to -0.1) | 0.031 |
| Wuwei | 2.47 (-5.04 to 10.58) | | | 0.53 | 0.12 (-2.3 to 2.6) | 0.924 |
| Wuxi | 0.42 (-0.26 to 1.11) | | | 0.229 | 0.69 (0.23 to 1.15) | 0.003 |
| Wuzhong | -0.63 (-3.16 to 1.98) | | | 0.635 | -0.35 (-1.58 to 0.89) | 0.579 |
| Xiangtan | 0.39 (-0.62 to 1.41) | | | 0.449 | 0.4 (-0.32 to 1.13) | 0.274 |
| Xiangyang | -0.35 (-1.67 to 1) | | | 0.613 | -0.27 (-1.44 to 0.92) | 0.655 |
| Xiaogan | 0.42 (-2.12 to 3.02) | | | 0.75 | 0.81 (-0.7 to 2.34) | 0.296 |
| Xilin Gol League | -4.53 (-9.9 to 1.15) | | | 0.116 | -1.05 (-2.63 to 0.55) | 0.198 |
| Xingtai | 0.82 (-0.32 to 1.97) | | | 0.162 | 0.79 (-0.05 to 1.63) | 0.064 |
| Xining | -1.67 (-4.32 to 1.04) | | | 0.225 | -0.97 (-1.75 to -0.18) | 0.016 |
| Xuancheng | 0.79 (-0.89 to 2.5) | | | 0.361 | -0.17 (-1.5 to 1.19) | 0.808 |
| Ya'an | 0.67 (-3.6 to 5.14) | | | 0.763 | 0.51 (-1.89 to 2.97) | 0.677 |
| Yancheng | 0.41 (-0.44 to 1.28) | | | 0.344 | 0.45 (-0.12 to 1.02) | 0.121 |
| Yangquan | 0.36 (-0.74 to 1.48) | | | 0.522 | -0.01 (-0.71 to 0.69) | 0.97 |
| Yangzhou | -1.09 (-3.17 to 1.03) | | | 0.312 | -0.45 (-1.81 to 0.92) | 0.518 |
| Yantai | 0.44 (-0.33 to 1.22) | | | 0.259 | 0.2 (-0.36 to 0.76) | 0.492 |
| Yibin | 0.24 (-0.87 to 1.37) | | | 0.67 | 0.36 (-0.5 to 1.22) | 0.415 |
| Yichang | 0.94 (0.04 to 1.84) | | | 0.041 | 0.94 (0.28 to 1.62) | 0.006 |
| Yichun | 3.66 (-0.68 to 8.19) | | | 0.1 | 2.23 (-0.49 to 5.04) | 0.11 |
| Yinchuan | 1.71 (-0.09 to 3.54) | | | 0.062 | 1 (0 to 2) | 0.05 |
| Yingkou | -0.57 (-1.6 to 0.47) | | | 0.279 | -0.49 (-1.27 to 0.3) | 0.227 |
| Yiyang | 1 (-0.98 to 3.02) | | | 0.323 | 1.22 (0.05 to 2.4) | 0.042 |
| Yizhou | 0.56 (-0.44 to 1.58) | | | 0.275 | 0.23 (-0.43 to 0.89) | 0.494 |
| Yongzhou | 0.91 (-2.06 to 3.96) | | | 0.553 | 1.1 (-0.97 to 3.21) | 0.302 |
| Yueyang | 0.08 (-1.43 to 1.61) | | | 0.921 | 0.29 (-0.58 to 1.17) | 0.518 |
| Yulin | -0.68 (-3.06 to 1.77) | | | 0.583 | 0.09 (-1.62 to 1.83) | 0.922 |
| Yuncheng | 0.28 (-0.78 to 1.35) | | | 0.606 | 0.16 (-0.57 to 0.89) | 0.674 |
| Yunfu | -12.53 (-30.02 to 9.33) | | | 0.241 | -8.76 (-21.7 to 6.32) | 0.242 |
| Yuxi | 2.74 (-0.16 to 5.73) | | | 0.065 | 1.29 (-0.58 to 3.19) | 0.177 |
| Zaozhuang | 0.99 (0.22 to 1.76) | | | 0.012 | 0.36 (-0.13 to 0.85) | 0.146 |
| Zhangjiajie | 2.4 (1.04 to 3.78) | | | 0.001 | 1.9 (0.93 to 2.88) | <0.001 |
| Zhangjiakou | 1.75 (-0.71 to 4.28) | | | 0.166 | 0.33 (-0.56 to 1.24) | 0.468 |
| Zhaotong | 3.5 (1.22 to 5.84) | | | 0.003 | 2.31 (0.88 to 3.76) | 0.002 |
| Zhenjiang | -0.82 (-2.34 to 0.71) | | | 0.292 | -0.32 (-1.36 to 0.73) | 0.548 |
| Zhongshan | 1.9 (-0.02 to 3.86) | | | 0.053 | 1.78 (0.41 to 3.17) | 0.011 |
| Zhongwei | 2.45 (-1.77 to 6.86) | | | 0.26 | 0.29 (-1.24 to 1.85) | 0.713 |
| Zhoushan | 1.25 (-0.82 to 3.36) | | | 0.239 | 0.53 (-0.9 to 1.98) | 0.47 |
| Zhuzhou | 0.29 (-0.86 to 1.45) | | | 0.626 | 0.39 (-0.46 to 1.26) | 0.371 |
| Zibo | 0.87 (0.27 to 1.47) | | | 0.004 | 0.58 (0.17 to 0.99) | 0.005 |
| Zigong | 0.75 (-0.35 to 1.85) | | | 0.182 | 0.27 (-0.58 to 1.12) | 0.535 |
| Ziyang | 0.51 (-2.27 to 3.37) | | | 0.721 | 0.48 (-1.4 to 2.39) | 0.619 |
| **Adjusted analyses**^*^ | |  | |  |  |  |
| An'shan | -0.92 (-2.31 to 0.49) | | | 0.202 | -0.37 (-1.44 to 0.71) | 0.500 |
| Anyang | -0.04 (-0.56 to 0.48) | | | 0.871 | 0.13 (-0.22 to 0.48) | 0.469 |
| Baicheng | -2.02 (-4.64 to 0.68) | | | 0.142 | -1.64 (-3.74 to 0.51) | 0.136 |
| Baise | -0.49 (-3.11 to 2.2) | | | 0.718 | -0.11 (-2.18 to 2) | 0.918 |
| Baiyin | -2.38 (-4.58 to -0.13) | | | 0.039 | -0.82 (-1.49 to -0.14) | 0.019 |
| Baoji | 0.61 (0.18 to 1.04) | | | 0.005 | 0.39 (0.09 to 0.69) | 0.011 |
| Baoshan | -0.35 (-4.36 to 3.83) | | | 0.867 | 0.87 (-1.49 to 3.28) | 0.473 |
| Baotou | 0.86 (-0.09 to 1.82) | | | 0.077 | 0.37 (-0.06 to 0.8) | 0.095 |
| Bayannur | -1.02 (-2.8 to 0.79) | | | 0.269 | -0.29 (-0.88 to 0.31) | 0.345 |
| Bazhong | -0.24 (-2.66 to 2.24) | | | 0.849 | 0.46 (-1.33 to 2.28) | 0.617 |
| Beihai | 2.84 (-2.76 to 8.76) | | | 0.327 | 0.48 (-4.17 to 5.36) | 0.842 |
| Bengbu | 0.12 (-1.96 to 2.26) | | | 0.908 | -0.06 (-1.28 to 1.18) | 0.923 |
| Benxi | -1.42 (-2.42 to -0.41) | | | 0.006 | -1.53 (-2.3 to -0.76) | <0.001 |
| Binzhou | 0.26 (-0.89 to 1.42) | | | 0.66 | 0.28 (-0.5 to 1.08) | 0.479 |
| Bozhou | 0.81 (-1.04 to 2.68) | | | 0.394 | 0.4 (-0.93 to 1.74) | 0.56 |
| Changde | 1.87 (0.57 to 3.19) | | | 0.005 | 1.67 (0.62 to 2.73) | 0.002 |
| Changsha | 0.03 (-1.17 to 1.25) | | | 0.964 | 1.27 (0.04 to 2.51) | 0.043 |
| Changzhi | 1.74 (0.03 to 3.47) | | | 0.046 | 0.72 (-0.44 to 1.9) | 0.225 |
| Changzhou | 1.12 (0.38 to 1.88) | | | 0.003 | 0.78 (0.26 to 1.31) | 0.003 |
| Chaoyang | -0.56 (-1.86 to 0.76) | | | 0.408 | -0.44 (-1.3 to 0.43) | 0.317 |
| Chengde | -0.38 (-2.89 to 2.2) | | | 0.77 | -0.18 (-1.17 to 0.81) | 0.721 |
| Chengdu | 0.94 (-0.29 to 2.18) | | | 0.135 | 0.7 (-0.11 to 1.51) | 0.09 |
| Chenzhou | 0.15 (-1.59 to 1.93) | | | 0.866 | 0.35 (-0.87 to 1.59) | 0.572 |
| Chifeng | 0.86 (-0.54 to 2.29) | | | 0.23 | 0.67 (-0.06 to 1.41) | 0.073 |
| Chizhou | 2.19 (-1.82 to 6.36) | | | 0.29 | 1.03 (-1.49 to 3.62) | 0.427 |
| Chongqing | -0.03 (-0.75 to 0.7) | | | 0.939 | 0.06 (-0.47 to 0.59) | 0.834 |
| Chongzuo | 0.99 (-4.24 to 6.51) | | | 0.717 | 0.57 (-3.69 to 5.01) | 0.798 |
| Chuzhou | 0.59 (-0.91 to 2.12) | | | 0.441 | 0.48 (-0.64 to 1.61) | 0.401 |
| Dalian | -0.7 (-1.8 to 0.41) | | | 0.216 | -0.95 (-1.76 to -0.14) | 0.022 |
| Dandong | 2.91 (1.04 to 4.8) | | | 0.002 | 2.15 (0.9 to 3.43) | <0.001 |
| Datong | 0.36 (-0.95 to 1.7) | | | 0.588 | 0.15 (-0.58 to 0.88) | 0.691 |
| Daxinganling | 0.36 (-1.94 to 2.71) | | | 0.762 | 0.44 (-1.42 to 2.33) | 0.645 |
| Dazhou | -0.79 (-1.99 to 0.42) | | | 0.198 | -0.72 (-1.61 to 0.18) | 0.117 |
| Dezhou | -0.12 (-0.73 to 0.48) | | | 0.688 | -0.14 (-0.55 to 0.26) | 0.485 |
| Dongying | 0.22 (-0.98 to 1.43) | | | 0.723 | 0.22 (-0.62 to 1.06) | 0.612 |
| Erdos | 2.21 (-0.09 to 4.55) | | | 0.06 | 0.19 (-0.47 to 0.85) | 0.572 |
| Fangchenggang | 6.5 (0.48 to 12.89) | | | 0.034 | 6.64 (1.45 to 12.1) | 0.012 |
| Fushun | -1.14 (-2.02 to -0.27) | | | 0.011 | -0.51 (-1.12 to 0.1) | 0.101 |
| Guangyuan | -3.23 (-5.46 to -0.95) | | | 0.006 | -1.12 (-2.23 to 0) | 0.05 |
| Guangzhou | -0.08 (-1.44 to 1.3) | | | 0.909 | 0.26 (-0.7 to 1.23) | 0.593 |
| Guigang | -1.59 (-5.89 to 2.92) | | | 0.484 | -0.84 (-3.82 to 2.23) | 0.586 |
| Guyuan | -2.99 (-9.27 to 3.72) | | | 0.374 | -1.62 (-3.95 to 0.76) | 0.181 |
| Haikou | 3.25 (-2.14 to 8.95) | | | 0.243 | 2.71 (-1.34 to 6.93) | 0.193 |
| Handan | 0.08 (-0.51 to 0.68) | | | 0.787 | -0.26 (-0.69 to 0.17) | 0.238 |
| Hangzhou | -0.23 (-0.93 to 0.47) | | | 0.515 | -0.21 (-0.71 to 0.3) | 0.419 |
| Hefei | 4.03 (1.82 to 6.29) | | | <0.001 | 2.7 (1.06 to 4.36) | <0.001 |
| Hegang | 1.2 (-0.47 to 2.91) | | | 0.161 | 0.8 (-0.52 to 2.13) | 0.236 |
| Heihe | -0.3 (-4.63 to 4.23) | | | 0.896 | 0.32 (-2.26 to 2.97) | 0.809 |
| Hengshui | -0.01 (-3.58 to 3.68) | | | 0.994 | 0.61 (-1.65 to 2.91) | 0.601 |
| Hengyang | 0.38 (-1.29 to 2.07) | | | 0.659 | 0.09 (-1.2 to 1.39) | 0.895 |
| Hetian | -1.12 (-2.64 to 0.42) | | | 0.155 | -0.01 (-0.76 to 0.75) | 0.983 |
| Heze | 0.38 (-0.3 to 1.08) | | | 0.276 | 0.38 (-0.09 to 0.86) | 0.113 |
| Hezhou | -1.07 (-4.39 to 2.38) | | | 0.54 | -0.07 (-2.49 to 2.42) | 0.958 |
| Hinggan League | -0.48 (-3.08 to 2.18) | | | 0.719 | -0.5 (-1.87 to 0.9) | 0.481 |
| Hohhot | 0.63 (-0.96 to 2.25) | | | 0.44 | 0.25 (-0.4 to 0.91) | 0.444 |
| Huai'an | 0.04 (-0.9 to 0.98) | | | 0.94 | 0.05 (-0.59 to 0.7) | 0.88 |
| Huaibei | -1.02 (-2.37 to 0.35) | | | 0.143 | -0.72 (-1.62 to 0.18) | 0.118 |
| Huaihua | 0.16 (-1.63 to 1.99) | | | 0.86 | 0.35 (-0.81 to 1.52) | 0.557 |
| Huainan | 0.35 (-0.58 to 1.28) | | | 0.464 | -0.11 (-0.66 to 0.44) | 0.696 |
| Huangshan | -0.46 (-3.24 to 2.39) | | | 0.748 | 0.14 (-1.98 to 2.3) | 0.9 |
| Huludao | 0.98 (0.02 to 1.94) | | | 0.045 | 0.48 (-0.14 to 1.11) | 0.133 |
| Hulunbeier | 0.99 (-1.53 to 3.57) | | | 0.444 | 0.46 (-0.62 to 1.55) | 0.409 |
| Jiaxing | 0.46 (-0.33 to 1.26) | | | 0.253 | 0.26 (-0.31 to 0.84) | 0.371 |
| Jiayuguan | -1.7 (-4.19 to 0.85) | | | 0.19 | -0.96 (-1.86 to -0.05) | 0.04 |
| Jilin | -0.08 (-0.79 to 0.64) | | | 0.836 | 0.18 (-0.36 to 0.73) | 0.516 |
| Jinan | 0.34 (-0.25 to 0.93) | | | 0.265 | 0.03 (-0.33 to 0.4) | 0.862 |
| Jincheng | 0.86 (-0.54 to 2.29) | | | 0.231 | 0.39 (-0.54 to 1.33) | 0.41 |
| Jinhua | -0.04 (-1.41 to 1.36) | | | 0.957 | -0.08 (-1.16 to 1.02) | 0.888 |
| Jining | 0.36 (-0.27 to 1) | | | 0.262 | 0.27 (-0.13 to 0.68) | 0.186 |
| Jinzhong | 0.14 (-1.1 to 1.41) | | | 0.821 | -0.01 (-0.81 to 0.79) | 0.972 |
| Jinzhou | -0.53 (-1.28 to 0.24) | | | 0.176 | -0.34 (-0.94 to 0.27) | 0.28 |
| Jixi | 1.07 (-1.18 to 3.37) | | | 0.355 | 0.27 (-1.39 to 1.95) | 0.752 |
| Karamay | 40.54 (-28.25 to 175.29) | | | 0.324 | 4.78 (-22.61 to 41.86) | 0.763 |
| Kashi | -0.23 (-0.83 to 0.38) | | | 0.455 | -0.07 (-0.23 to 0.09) | 0.399 |
| Kiamusze | 4.51 (-5.8 to 15.94) | | | 0.406 | 3.29 (-3.25 to 10.28) | 0.334 |
| Kunming | 1.39 (-1.14 to 3.98) | | | 0.283 | 1.25 (-0.15 to 2.67) | 0.08 |
| Laibin | 2.99 (-0.63 to 6.75) | | | 0.108 | 2.53 (-0.42 to 5.57) | 0.094 |
| Laiwu | 1.44 (0.4 to 2.49) | | | 0.007 | 1.21 (0.46 to 1.97) | 0.002 |
| Langfang | 1.61 (0.26 to 2.97) | | | 0.019 | 1.31 (0.3 to 2.33) | 0.011 |
| Lanzhou | -2.95 (-4.88 to -0.97) | | | 0.004 | -1.05 (-1.63 to -0.45) | 0.001 |
| Lianyungang | 0.7 (-1.71 to 3.17) | | | 0.571 | 0.55 (-0.83 to 1.95) | 0.435 |
| Liaocheng | 0.29 (-0.26 to 0.84) | | | 0.308 | 0.05 (-0.33 to 0.44) | 0.785 |
| Liaoyang | -1.5 (-2.23 to -0.76) | | | <0.001 | -1.23 (-1.81 to -0.65) | <0.001 |
| Liaoyuan | -0.06 (-1.96 to 1.87) | | | 0.949 | -0.45 (-2 to 1.14) | 0.579 |
| Lijiang | 5.87 (-6.39 to 19.74) | | | 0.364 | -0.23 (-7.01 to 7.05) | 0.95 |
| Linfen | 1.26 (0.16 to 2.37) | | | 0.025 | 1.01 (0.14 to 1.88) | 0.024 |
| Lishui | -1.04 (-3.23 to 1.2) | | | 0.36 | -0.51 (-2.29 to 1.31) | 0.581 |
| Liu'an | 0.41 (-1.37 to 2.23) | | | 0.653 | 0.52 (-0.7 to 1.75) | 0.41 |
| Liuzhou | 0.98 (-0.92 to 2.91) | | | 0.316 | 0.74 (-0.59 to 2.09) | 0.275 |
| Longnan | 98.6 (-9.29 to 334.83) | | | 0.089 | 46.7 (-12.33 to 145.5) | 0.148 |
| Loudi | 0.83 (-0.97 to 2.67) | | | 0.368 | 0.76 (-0.51 to 2.05) | 0.241 |
| Lvliang | 3.39 (-0.75 to 7.7) | | | 0.111 | 1.83 (-0.21 to 3.91) | 0.08 |
| Ma'anshan | -1.78 (-3.06 to -0.47) | | | 0.008 | -1.11 (-2.08 to -0.13) | 0.026 |
| Maoming | 0.93 (-4.02 to 6.13) | | | 0.719 | 5.55 (1.31 to 9.97) | 0.01 |
| Mianyang | -2.19 (-10.17 to 6.5) | | | 0.612 | -2.34 (-8.43 to 4.16) | 0.473 |
| Mudanjiang | 1.25 (-3.4 to 6.12) | | | 0.605 | 0.9 (-2.4 to 4.32) | 0.596 |
| Nanchang | 3.82 (2.43 to 5.23) | | | <0.001 | 2.6 (1.62 to 3.6) | <0.001 |
| Nanchong | -1.94 (-2.98 to -0.9) | | | <0.001 | -1.29 (-2 to -0.57) | <0.001 |
| Nanjing | 1.65 (0.5 to 2.81) | | | 0.005 | 1.47 (0.68 to 2.26) | <0.001 |
| Nanning | 5.41 (2.07 to 8.86) | | | 0.001 | 4.05 (1.97 to 6.17) | <0.001 |
| Nantong | 0.3 (-0.33 to 0.94) | | | 0.352 | -0.02 (-0.48 to 0.43) | 0.915 |
| Ningbo | 0.02 (-0.77 to 0.81) | | | 0.963 | 0.15 (-0.41 to 0.72) | 0.597 |
| Panjin | -0.25 (-1.27 to 0.77) | | | 0.626 | 0.17 (-0.65 to 1.01) | 0.681 |
| Pu'er | -0.89 (-3.52 to 1.81) | | | 0.515 | 0.02 (-2.06 to 2.14) | 0.986 |
| Qingyang | 0.64 (-2.05 to 3.39) | | | 0.645 | -1.2 (-2.52 to 0.14) | 0.079 |
| Qingyuan | 0.82 (-0.53 to 2.18) | | | 0.236 | 1.14 (0.22 to 2.07) | 0.015 |
| Qinhuangdao | -0.45 (-1.7 to 0.82) | | | 0.487 | -0.52 (-1.35 to 0.32) | 0.222 |
| Qinzhou | 0.44 (-3.21 to 4.22) | | | 0.817 | -1.35 (-4.23 to 1.61) | 0.368 |
| Qiqihar | 1.78 (-5.45 to 9.56) | | | 0.639 | -1.62 (-6.71 to 3.74) | 0.546 |
| Qujing | 0.42 (-1.58 to 2.45) | | | 0.685 | 0.6 (-0.79 to 2) | 0.399 |
| Quzhou | 1.19 (-0.12 to 2.51) | | | 0.075 | -0.67 (-1.64 to 0.31) | 0.182 |
| Sanya | 4.89 (-2.34 to 12.65) | | | 0.191 | 3.55 (-1.67 to 9.06) | 0.187 |
| Shangrao | 1.69 (-1.08 to 4.55) | | | 0.235 | 2.73 (0.99 to 4.49) | 0.002 |
| Shantou | -1.31 (-3.63 to 1.07) | | | 0.278 | -1.01 (-2.73 to 0.73) | 0.253 |
| Shaoxing | -0.12 (-1.07 to 0.83) | | | 0.8 | -0.24 (-0.92 to 0.45) | 0.493 |
| Shaoyang | -0.5 (-1.97 to 0.98) | | | 0.504 | -0.26 (-1.41 to 0.91) | 0.667 |
| Shenyang | -0.24 (-0.74 to 0.28) | | | 0.365 | -0.18 (-0.59 to 0.24) | 0.404 |
| Shiyan | 0.14 (-1.59 to 1.89) | | | 0.877 | 0.06 (-1.18 to 1.32) | 0.921 |
| Shizuishan | 0.18 (-1.93 to 2.33) | | | 0.87 | -0.58 (-1.39 to 0.22) | 0.157 |
| Shuozhou | 0.41 (-1.38 to 2.25) | | | 0.654 | 0.29 (-0.85 to 1.44) | 0.62 |
| Siping | -1.66 (-3.06 to -0.24) | | | 0.022 | -0.6 (-1.69 to 0.5) | 0.282 |
| Suzhou | 0.05 (-0.41 to 0.53) | | | 0.557 | 0.1 (-0.29 to 0.49) | 0.236 |
| Suzhou | 0.42 (-0.97 to 1.83) | | | 0.557 | -0.67 (-1.77 to 0.44) | 0.236 |
| Taian | 1.14 (0.43 to 1.85) | | | 0.002 | 0.81 (0.31 to 1.3) | 0.001 |
| Taiyuan | 4.2 (0.29 to 8.26) | | | 0.037 | 1.75 (-1.08 to 4.65) | 0.23 |
| Taizhou | -0.04 (-3.61 to 3.66) | | | 0.493 | 0.1 (-2.3 to 2.56) | 0.902 |
| Taizhou | 0.74 (-1.37 to 2.9) | | | 0.493 | 0.09 (-1.29 to 1.49) | 0.902 |
| Tangshan | 0.17 (-0.57 to 0.92) | | | 0.651 | 0.45 (-0.01 to 0.91) | 0.053 |
| Tianjin | 0.14 (-0.5 to 0.78) | | | 0.666 | 0.2 (-0.26 to 0.66) | 0.394 |
| Tieling | -0.03 (-0.89 to 0.84) | | | 0.947 | -0.07 (-0.79 to 0.66) | 0.859 |
| Tonghua | -1.22 (-3.94 to 1.56) | | | 0.386 | -0.74 (-2.49 to 1.04) | 0.414 |
| Tongliao | 0.23 (-1.47 to 1.96) | | | 0.796 | 0.39 (-0.5 to 1.29) | 0.39 |
| Tongling | 0.31 (-1.51 to 2.17) | | | 0.739 | 0.03 (-1.33 to 1.41) | 0.962 |
| Turpan | 1.49 (0.29 to 2.71) | | | 0.015 | 0.45 (0.06 to 0.84) | 0.026 |
| Ulanqab | -0.2 (-1.92 to 1.56) | | | 0.825 | 0.39 (-0.29 to 1.08) | 0.261 |
| Weifang | 1.2 (0.59 to 1.81) | | | <0.001 | 0.7 (0.27 to 1.13) | 0.002 |
| Weihai | -0.01 (-1.31 to 1.31) | | | 0.99 | -0.58 (-1.45 to 0.3) | 0.199 |
| Wenzhou | -1.31 (-3.01 to 0.41) | | | 0.136 | -0.42 (-1.53 to 0.7) | 0.46 |
| Wuhai | 1.04 (-0.49 to 2.6) | | | 0.183 | 0.31 (-0.27 to 0.89) | 0.299 |
| Wuhan | 1.34 (0.51 to 2.18) | | | <0.001 | 0.78 (0.22 to 1.34) | 0.006 |
| Wuhu | -1.11 (-2.07 to -0.15) | | | 0.024 | -0.51 (-1.21 to 0.2) | 0.158 |
| Wuwei | 0.75 (-3.53 to 5.23) | | | 0.736 | -0.11 (-1.24 to 1.03) | 0.845 |
| Wuxi | 0.26 (-0.41 to 0.93) | | | 0.456 | 0.46 (-0.01 to 0.93) | 0.053 |
| Wuzhong | -0.81 (-3.05 to 1.48) | | | 0.484 | -0.08 (-1.19 to 1.03) | 0.881 |
| Xiangtan | -0.46 (-1.72 to 0.8) | | | 0.47 | -0.49 (-1.44 to 0.48) | 0.323 |
| Xiangyang | -0.36 (-1.2 to 0.49) | | | 0.406 | -0.78 (-1.54 to -0.02) | 0.046 |
| Xiaogan | 0.21 (-2 to 2.48) | | | 0.853 | -0.1 (-1.4 to 1.21) | 0.881 |
| Xilin Gol League | -5.47 (-11.09 to 0.5) | | | 0.072 | -1.2 (-2.8 to 0.43) | 0.149 |
| Xingtai | 0.2 (-0.63 to 1.03) | | | 0.646 | -0.04 (-0.64 to 0.57) | 0.896 |
| Xining | -0.14 (-2.59 to 2.37) | | | 0.912 | -0.32 (-1.07 to 0.43) | 0.4 |
| Xuancheng | 2.08 (0.4 to 3.8) | | | 0.015 | 1.01 (-0.36 to 2.39) | 0.149 |
| Ya'an | -1.82 (-5.36 to 1.85) | | | 0.328 | -1.27 (-3.3 to 0.81) | 0.232 |
| Yancheng | 1.29 (0.46 to 2.12) | | | 0.002 | 0.9 (0.34 to 1.46) | 0.002 |
| Yangquan | 0.34 (-0.86 to 1.56) | | | 0.579 | 0.12 (-0.59 to 0.84) | 0.739 |
| Yangzhou | -0.4 (-2.87 to 2.14) | | | 0.756 | 0.49 (-1.29 to 2.31) | 0.591 |
| Yantai | 0.6 (-0.23 to 1.44) | | | 0.16 | 0.32 (-0.29 to 0.92) | 0.304 |
| Yibin | 0.86 (-0.13 to 1.87) | | | 0.089 | 0.75 (-0.03 to 1.54) | 0.059 |
| Yichang | 0.66 (-0.18 to 1.51) | | | 0.125 | 0.62 (-0.05 to 1.28) | 0.07 |
| Yichun | -0.03 (-2.32 to 2.3) | | | 0.977 | 0.73 (-0.87 to 2.35) | 0.375 |
| Yinchuan | 3.09 (1.28 to 4.93) | | | 0.001 | 1.63 (0.67 to 2.59) | 0.001 |
| Yingkou | -0.81 (-1.91 to 0.29) | | | 0.147 | -0.62 (-1.44 to 0.21) | 0.145 |
| Yiyang | 3.26 (1.04 to 5.52) | | | 0.004 | 2.88 (1.49 to 4.3) | <0.001 |
| Yizhou | 0.93 (-0.06 to 1.93) | | | 0.066 | 0.5 (-0.16 to 1.16) | 0.135 |
| Yongzhou | -0.96 (-4.04 to 2.22) | | | 0.55 | -0.1 (-2.39 to 2.25) | 0.932 |
| Yueyang | -1.6 (-3.24 to 0.07) | | | 0.06 | -1.01 (-1.94 to -0.06) | 0.037 |
| Yulin | 0.62 (-2.07 to 3.39) | | | 0.655 | 0.92 (-1.19 to 3.07) | 0.398 |
| Yuncheng | 0.38 (-0.47 to 1.24) | | | 0.384 | 0.43 (-0.14 to 1.01) | 0.14 |
| Yunfu | -17.44 (-35.41 to 5.53) | | | 0.128 | -11.28 (-25.75 to 6) | 0.189 |
| Yuxi | 1.49 (-0.71 to 3.75) | | | 0.187 | 0.88 (-0.59 to 2.39) | 0.243 |
| Zaozhuang | 1.41 (0.62 to 2.21) | | | <0.001 | 0.73 (0.22 to 1.24) | 0.005 |
| Zhangjiajie | 2.17 (0.76 to 3.59) | | | 0.002 | 1.77 (0.7 to 2.84) | 0.001 |
| Zhangjiakou | 1.01 (-1.36 to 3.43) | | | 0.408 | 0.37 (-0.47 to 1.23) | 0.389 |
| Zhaotong | 4.89 (2.65 to 7.19) | | | <0.001 | 3.38 (1.83 to 4.96) | <0.001 |
| Zhenjiang | -1.1 (-2.55 to 0.38) | | | 0.145 | -0.55 (-1.6 to 0.52) | 0.312 |
| Zhongshan | 1.09 (-0.81 to 3.02) | | | 0.264 | 1.09 (-0.35 to 2.55) | 0.14 |
| Zhongwei | 3.79 (-0.13 to 7.86) | | | 0.059 | 1.06 (-0.37 to 2.5) | 0.148 |
| Zhoushan | 2.14 (0.13 to 4.18) | | | 0.037 | 0.89 (-0.57 to 2.37) | 0.234 |
| Zhuzhou | 0.06 (-1.22 to 1.35) | | | 0.932 | -0.04 (-1.04 to 0.97) | 0.941 |
| Zibo | 0.98 (0.44 to 1.53) | | | <0.001 | 0.75 (0.38 to 1.11) | <0.001 |
| Zigong | 0.23 (-0.38 to 0.83) | | | 0.465 | 0.29 (-0.18 to 0.77) | 0.229 |
| Ziyang | 1.04 (-1.25 to 3.38) | | | 0.376 | 0.52 (-1.07 to 2.13) | 0.527 |

PM_2.5_, particulate matter ≤2.5 μm in aerodynamic diameter; PM_10_, particulate matter ≤10 μm in aerodynamic diameter.

^*^ Estimates were adjusted for temperature, relative humidity, calendar time, day of the week, and public holiday.
