# Supplementary material for: Ambient particulate matter pollution and adult hospital admissions for pneumonia in urban China: A national time series analysis for 2014 through 2017
Source: PLoS Med. 2019 Dec 31;16(12):e1003010. doi: 10.1371/journal.pmed.1003010 (PMC6938337; doi:10.1371/journal.pmed.1003010)
Supplement: S3 Table — (DOCX) [file pmed.1003010.s003.docx]

**S3 Table.** Regional-average percentage increase with 95% confidence interval in daily hospital admissions for pneumonia associated with a 10 μg/m^3^ increase in PM_2.5_ and PM_10_ concentrations (lag 0-2) in 184 Chinese cities, 2014–2017.

| **Region** | **PM_2.5_** | |  | **PM_10_** |  |
| --- | --- | --- | --- | --- | --- |
|  | Percentage increase (95% confidence interval) | | *P* value | Percentage increase (95% confidence interval) | *P* value |
| **Unadjusted analyses** | |  |  |  |  |
| East | 0.22 (0.02 to 0.43) | | 0.033 | 0.14 (0.01 to 0.28) | 0.041 |
| Middle-south | 0.88 (0.47 to 1.29) | | < 0.001 | 0.86 (0.56 to 1.16) | < 0.001 |
| Southwest | 0.09 (-0.64 to 0.82) | | 0.815 | 0.24 (-0.16 to 0.65) | 0.231 |
| Northwest | -0.23 (-0.98 to 0.53) | | 0.549 | -0.45 (-0.83 to -0.08) | 0.018 |
| North | 0.19 (-0.03 to 0.41) | | 0.087 | 0.07 (-0.08 to 0.22) | 0.366 |
| Northeast | -0.39 (-0.72 to -0.05) | | 0.025 | -0.27 (-0.53 to -0.01) | 0.041 |
| **Adjusted analyses**^*^ |  | |  |  |  |
| East | 0.41 (0.20 to 0.63) | | < 0.001 | 0.23 (0.08 to 0.38) | 0.002 |
| Middle-south | 0.66 (0.23 to 1.10) | | 0.003 | 0.69 (0.33 to 1.06) | < 0.001 |
| Southwest | 0.14 (-0.56 to 0.85) | | 0.689 | 0.25 (-0.24 to 0.73) | 0.318 |
| Northwest | 0.09 (-0.70 to 0.89) | | 0.875 | -0.14 (-0.46 to 0.18) | 0.384 |
| North | 0.44 (0.21 to 0.67) | | < 0.001 | 0.24 (0.10 to 0.38) | < 0.001 |
| Northeast | -0.36 (-0.73 to 0.01) | | 0.053 | -0.23 (-0.53 to 0.07) | 0.128 |

PM_2.5_, particulate matter ≤2.5 μm in aerodynamic diameter; PM_10_, particulate matter ≤10 μm in aerodynamic diameter.

^*^ Estimates were adjusted for temperature, relative humidity, calendar time, day of the week, and public holiday.
