# Supplementary material for: Ambient particulate matter pollution and adult hospital admissions for pneumonia in urban China: A national time series analysis for 2014 through 2017
Source: PLoS Med. 2019 Dec 31;16(12):e1003010. doi: 10.1371/journal.pmed.1003010 (PMC6938337; doi:10.1371/journal.pmed.1003010)
Supplement: S4 Table — (DOCX) [file pmed.1003010.s004.docx]

**S4 Table.** National-average percentage increase with 95% confidence interval in daily hospital admissions for pneumonia associated with a 10 μg/m^3^ increase in PM_2.5_ and PM_10_ concentrations (lag 0-2) in two-pollutant models in 184 Chinese cities, 2014–2017.

| **Variables** | **PM_2.5_** | |  | | | **PM_10_** |  | |
| --- | --- | --- | --- | --- | --- | --- | --- | --- |
|  | Percentage increase (95% confidence interval) | | *P* value | | | Percentage increase (95% confidence interval) | *P* value | |
| **Unadjusted analyses** | |  | |  | |  |  | |
| Adjusted for SO_2_ | 0.09 (-0.06 to 0.23) | | | | 0.234 | 0.05 (-0.05 to 0.15) | | 0.373 |
| Adjusted for NO_2_ | -0.07 (-0.21 to 0.08) | | | | 0.370 | -0.06 (-0.16 to 0.04) | | 0.241 |
| Adjusted for CO | 0.02 (-0.13 to 0.18) | | | | 0.772 | 0.03 (-0.08 to 0.14) | | 0.552 |
| Adjusted for O_3_ | 0.20 (0.05 to 0.34) | | | | 0.008 | 0.13 (0.02 to 0.23) | | 0.017 |
| **Adjusted analyses**^*^ |  | | |  | |  |  | |
| Adjusted for SO_2_ | 0.22 (0.07 to 0.37) | | | | 0.006 | 0.12 (0.02 to 0.22) | | 0.015 |
| Adjusted for NO_2_ | 0.10 (-0.05 to 0.25) | | | | 0.271 | 0.04 (-0.06 to 0.14) | | 0.431 |
| Adjusted for CO | 0.12 (-0.03 to 0.27) | | | | 0.127 | 0.06 (-0.04 to 0.16) | | 0.256 |
| Adjusted for O_3_ | 0.31 (0.16 to 0.46) | | | | < 0.001 | 0.20 (0.09 to 0.30) | | < 0.001 |

PM_2.5_, particulate matter ≤2.5 μm in aerodynamic diameter; PM_10_, particulate matter ≤10 μm in aerodynamic diameter.

^*^ Estimates were also adjusted for temperature, relative humidity, calendar time, day of the week, and public holiday.
