# Supplementary material for: Ambient particulate matter pollution and adult hospital admissions for pneumonia in urban China: A national time series analysis for 2014 through 2017
Source: PLoS Med. 2019 Dec 31;16(12):e1003010. doi: 10.1371/journal.pmed.1003010 (PMC6938337; doi:10.1371/journal.pmed.1003010)
Supplement: S5 Table — (DOCX) [file pmed.1003010.s005.docx]

**S5 Table:** Results of sensitivity analyses.

| **Variables** | | **PM_2.5_** | | |  | | **PM_10_** | |  |
| --- | --- | --- | --- | --- | --- | --- | --- | --- | --- |
|  | | Percentage increase (95% confidence interval) | | | *P* value | | Percentage increase (95% confidence interval) | | *P* value |
| **Unadjusted analyses** | | |  |  | |  | |  | |
| Degree of freedom for calendar time | |  | | |  | |  | |  |
| 6 | | 0.24 (0.11 to 0.37) | | | < 0.001 | | 0.17 (0.06 to 0.27) | | 0.002 |
| 7 | | 0.23 (0.08 to 0.37) | | | 0.002 | | 0.15 (0.04 to 0.25) | | 0.005 |
| 8 | | 0.15 (0.03 to 0.28) | | | 0.015 | | 0.07 (-0.02 to 0.16) | | 0.103 |
| 9 | | 0.16 (0.03 to 0.28) | | | 0.015 | | 0.08 (-0.01 to 0.16) | | 0.091 |
| 10 | | 0.24 (0.09 to 0.38) | | | 0.001 | | 0.10 (0.01 to 0.21) | | 0.040 |
| Dataset | |  | | |  | |  | |  |
| 78 cities with 3-year | | 0.21 (-0.06 to 0.49) | | | 0.134 | | 0.16 (0.03 to 0.29) | | 0.013 |
| 106 cities with 4-year data | | 0.23 (0.05 to 0.40) | | | 0.011 | | 0.10 (-0.07 to 0.28) | | 0.236 |
| Spline function | |  | | |  | |  | |  |
| Penalized spline function | | 0.23 (0.10 to 0.36) | | | < 0.001 | | 0.16 (0.06 to 0.26) | | 0.001 |
| Coverage of population by UEBMI (%) | |  | | |  | |  | |  |
| < 20 | | 0.27 (0.10 to 0.44) | | | 0.002 | | 0.18 (0.07 to 0.30) | | 0.002 |
| ≥ 20 | | 0.15 (-0.10 to 0.40) | | | 0.230 | | 0.08 (-0.10 to 0.26) | | 0.391 |
| Exclusion of cities with ≤2 monitors | | 0.22 (0.08 to 0.36) | | | 0.008 | | 0.16 (0.05 to 0.26) | | 0.003 |
| PM_10_ (μg/m^3^) | |  | | |  | |  | |  |
| < 100 μg/m^3^ | | ― | | |  | | 0.04 (-0.11 to 0.19) | | 0.754 |
| ≥ 100 μg/m^3^ | | ― | | |  | | 0.31 (0.09 to 0.53) | | < 0.001 |
| Adjusting for hospitalization of influenza | | 0.22 (0.07 to 0.37) | | | 0.007 | | 0.14 (0.02 to 0.24) | | 0.012 |
| **Adjusted analyses**^*^ |  | | |  | |  | |  | |
| Degree of freedom for calendar time | |  | | |  | |  | |  |
| 6 | | 0.32 (0.17 to 0.47) | | | < 0.001 | | 0.20 (0.10 to 0.30) | | < 0.001 |
| 7 | | 0.31 (0.15 to 0.46) | | | < 0.001 | | 0.19 (0.11 to 0.30) | | < 0.001 |
| 8 | | 0.26 (0.13 to 0.39) | | | < 0.001 | | 0.20 (0.10 to 0.30) | | < 0.001 |
| 9 | | 0.27 (0.14 to 0.40) | | | < 0.001 | | 0.15 (0.06 to 0.24) | | 0.001 |
| 10 | | 0.25 (0.12 to 0.38) | | | < 0.001 | | 0.13 (0.04 to 0.22) | | 0.015 |
| Dataset | |  | | |  | |  | |  |
| 78 cities with 3-year | | 0.28 (0 to 0.56) | | | 0.045 | | 0.13 (-0.07 to 0.34) | | 0.137 |
| 106 cities with 4-year data | | 0.37 (0.20 to 0.56) | | | < 0.001 | | 0.24 (0.11 to 0.37) | | < 0.001 |
| Spline function | |  | | |  | |  | |  |
| Penalized spline function | | 0.31 (0.17 to 0.45) | | | < 0.001 | | 0.22 (0.12 to 0.32) | | < 0.001 |
| Coverage of population by UEBMI (%) | |  | | |  | |  | |  |
| < 20 | | 0.29 (0.10 to 0.48) | | | < 0.001 | | 0.20 (0.07 to 0.33) | | < 0.001 |
| ≥ 20 | | 0.35 (0.09 to 0.61) | | | < 0.001 | | 0.19 (0.01 to 0.36) | | 0.038 |
| Exclusion of cities with ≤2 monitors | | 0.30 (0.15 to 0.46) | | | < 0.001 | | 0.21(0.10 to 0.32) | | < 0.001 |
| PM_10_ (μg/m^3^) | |  | | |  | |  | |  |
| < 100 μg/m^3^ | | ― | | |  | | 0.38 (0.18 to 0.57) | | < 0.001 |
| ≥ 100 μg/m^3^ | | ― | | |  | | 0.10 (-0.10 to 0.30) | | 0.368 |
| Adjusting for hospitalization of influenza | | 0.29 (0.13 to 0.45) | | | < 0.001 | | 0.18 (0.07 to 0.29) | | < 0.001 |

PM_2.5_, particulate matter ≤2.5 μm in aerodynamic diameter; PM_10_, particulate matter ≤10 μm in aerodynamic diameter.

^*^ Estimates were adjusted for temperature, relative humidity, calendar time, day of the week, and public holiday.
