# Supplementary material for: Ambient particulate matter pollution and adult hospital admissions for pneumonia in urban China: A national time series analysis for 2014 through 2017
Source: PLoS Med. 2019 Dec 31;16(12):e1003010. doi: 10.1371/journal.pmed.1003010 (PMC6938337; doi:10.1371/journal.pmed.1003010)
Supplement: S1 Appendix — (DOCX) [file pmed.1003010.s007.docx]

**Statistical Analyses Plan**

**20 April 2018**

1. **Objective**

To assess the associations between short-term exposure to ambient particulate matter (PM) pollution and daily hospital admissions for pneumonia in Chinese adults.

1. **Exposure**

City-specific daily PM_2.5_ (PM ≤2.5 μm in aerodynamic diameter) and PM_10_ (PM ≤10 μm in aerodynamic diameter) concentrations were obtained from the National Air Pollution Monitoring System.

1. **Outcome**

Daily counts of city-specific hospital admissions for pneumonia.

1. **Method**

The associations between PM and daily hospital admissions for pneumonia are estimated by a two-stage approach. In the first stage, quasi-Poisson regression model is fitted to estimate city-specific associations of hospital admissions for pneumonia associated with PM exposure. In the second stage, a random-effects meta-analysis is conducted to pool the city-specific estimates.

1. **Primary analysis**

In the first stage, confounding risk factors included in the quasi-Poisson regression model as follows:

- Long-term time trends: a natural cubic spline of calendar time with 7 degrees of freedom (*df*) per year;
- Relative humidity: a natural cubic spline of 3-day moving average relative humidity with 3 *df*;
- Temperature: a natural cubic spline of 3-day moving average temperature with 6 *df*;
- Day of the week and public holiday: indicator variables.

Consequently, the model is as shown below:

Log[E(Y_t_)] = α + β(PM) + day of the week + public holiday + *ns*(time, *df* = 7/per year) + *ns*(temperature, *df* = 6) + *ns*(relative humidity, *df* = 3)

Where E(Yt) is the expected number of pneumonia admissions on day t; β indicates the log-relative risk of pneumonia associated with a unit increase of PM concentrations; ns() indicates a natural cubic spline function; temperature and relative humidity indicate 3-day moving averages; public holiday and day of the week were included in the model as indicator variables.

**Primary statistics to report**: percentage change and 95% CI in pneumonia admissions per 10-μg/m^3^ increase in PM concentrations. Percentage change equals relative risk minus 1 and then multiplies by 100.

**Shape of association**

A natural cubic spline of PM will be used to investigate the shape association between PM and pneumonia admissions. Relative change and 95% CIs are plotted against PM.

**Potential Effect modification**

For cities’ characteristics, city-specific relative risk (and their CIs) as the outcome are meta-regressed on each continuous variable of city characteristics. City-level characteristics include annual-average PM levels, temperature and relative humidity, gross domestic product per capita, and coverage rates by the UEBMI.

Effect modification by individual characteristics are investigated by stratified analyses. The subgroup variables include sex, age groups (18–64, 65–74 and ≥75 years) and geographical region (north and south regions).

1. **Sensitivity analysis**

The following sensitivity analyses will be conducted:

- Assessing the potential confounding effects of gaseous pollutants (sulfur dioxide (SO_2_), nitrogen dioxide (NO_2_), carbon monoxide (CO), and ozone (O_3_)) using two-pollutant models;
- Assessing the effects of PM on pneumonia in cities with only 3- or 4-year data;
- Assessing the effects of PM on pneumonia in cities with different levels of population coverage by the UEBMI (<20% and ≥20%);
- Changing the *df* for time (6–10 per year);
- Using penalized spline functions for time and meteorological variables;
- Excluding cities with ≤2 monitoring stations.

1. **Proposed Main Tables & Figures**

**Figure 1 Locations of the cities included in the study.**

**Table 1 Summary statistics of health and environmental data during the study period.**

- Average and range of annual mean hospital admissions for pneumonia across the cities
- Average and range of annual mean relative humidity across the cities
- Average and range of annual mean temperature across the cities
- Average and range of annual mean PM_2.5_ and PM_10_ concentrations across the cities

**Table 2 Spearman correlation coefficients among the exposure variables across the cities.**

**Table 3 Primary analysis for the national-average associations between PM and pneumonia for different lag days.**

**Table 4 Stratified analyses for the associations between PM and pneumonia.**

- Subgroup variables: sex, age groups (18–64, 65–74 and ≥75 years) and geographical region (north and south regions).

**Figure 2 Shape of associations between PM and pneumonia.**

**Table 5 Potential effect modifications of the associations between PM and pneumonia by city-level characteristics**

- City-level characteristics: annual-average PM levels, temperature and relative humidity, gross domestic product per capita, and coverage rates by the UEBMI.

**Table 6 Results from the sensitivity analysis**

- Adjusting for gaseous pollutant (SO_2_, NO_2_, CO and O_3_ concentrations) in the primary models.
- Assessing the effects of PM on pneumonia in cities with only 3- or 4-year data
- Assessing the effects of PM on pneumonia in cities with different levels of population coverage by the UEBMI (<20% and ≥20%)
- Changing the *df* for time (6–10 per year)
- Using penalized spline functions for time and meteorological variables
- Excluding cities with ≤2 monitoring stations.
