# Supplementary material for: Ambient particulate matter pollution and adult hospital admissions for pneumonia in urban China: A national time series analysis for 2014 through 2017
Source: PLoS Med. 2019 Dec 31;16(12):e1003010. doi: 10.1371/journal.pmed.1003010 (PMC6938337; doi:10.1371/journal.pmed.1003010)
Supplement: Attachment — Submitted filename: Response to Editors and Reviewers.docx [file pmed.1003010.s008.docx]

**Responses to comments of editors and reviewers**

***Requests from the editors:***

1. Did your study have a prospective protocol or analysis plan? Please state this (either way) early in the Methods section.

a) If a prospective analysis plan (from your funding proposal, IRB or other ethics committee submission, study protocol, or other planning document written before analyzing the data) was used in designing the study, please include the relevant prospectively written document with your revised manuscript as a Supporting Information file to be published alongside your study, and cite it in the Methods section. A legend for this file should be included at the end of your manuscript.

b) If no such document exists, please make sure that the Methods section transparently describes when analyses were planned, and when/why any data-driven changes to analyses took place.

c) In either case, changes in the analysis—including those made in response to peer review comments—should be identified as such in the Methods section of the paper, with rationale.

*Response*

As requested, we attached the translation of our pre-specified statistical analysis plan in this revision, where majority of the detailed information, especially for the model specification, were already included in the manuscript. We stated in the methods that “The method and the model used in this study were designed before the analyses were conducted, and the pre-specified statistical analysis plan was present in the Supporting Information (**S1 Appendix**).” (lines 191-193)

2. Abstract Methods and Findings: Line 29: Please change 0.28 billion adults to a more intuitive number (perhaps report in millions instead of billions).

*Response*

We thank the Editors for this comment. We have changed the sentence as “Information on daily hospital admissions for pneumonia for 2014–2017 was collected from the database of Urban Employee Basic Medical Insurance, which covers 282.93 million adults.” (lines 32-33)

3. Abstract Methods and Findings: Please include the study design.

*Response*

We have indicated the study design as “A nationwide time-series study was conducted in China between 2014 and 2017.” (lines 31-32)

4. Abstract Methods and Findings: Please quantify the results of the association between PM and hospital admissions with p values.

*Response*

Done.

5. Abstract Methods and Findings: Please quantify the results of the effects of PM with higher temperatures/humidity and in elderly adults with 95% CIs and p values.

*Response*

Done.

6. Abstract Conclusions: Please avoid assertions of primacy (“As the first study in China to investigate…”).

*Response*

We thank the Editors for this comment. We have deleted this sentence.

7. Abstract Conclusions: Your study is observational and therefore causality cannot be inferred. Please remove language that implies causality (...may assist in understanding how PM causes lung-inflammation diseases.).

*Response*

We thank the Editors for this comment. We have deleted this sentence.

8. At this stage, we ask that you include a short, non-technical Author Summary of your research to make findings accessible to a wide audience that includes both scientists and non-scientists. The Author Summary should immediately follow the Abstract in your revised manuscript. This text is subject to editorial change and should be distinct from the scientific abstract. Please see our author guidelines for more information: https://journals.plos.org/plosmedicine/s/revising-your-manuscript#loc-author-summary.

*Response*

We have included an Author Summary in this revision.

9. Methods: Please provide details on the 184 cities included in the study, including criteria for the selection of cities and the years of the study period for each city.

*Response*

We thank the Editors for this comment. We have now clarified in the Method that “Total 184 Chinese cities were finally included in this study. Cities were selected according to the availability of daily hospital admission and air pollution data. Cities with only 1-year hospital admission records were excluded owing to the feasibility of model fit. Individuals’ detailed disease diagnosis (International Classification of Diseases (ICD) code and text of disease diagnosis) was required to identify pneumonia admissions. Cities with no information on disease diagnosis recorded in the database were also excluded. In addition, we applied additional rule to control the quality of the original data. We regressed the city-specific average numbers of pneumonia admission per day on the number of enrollers of the city. Cities with absolute value of standardized residuals > 3 were considered as the outliers, and were removed from the analysis. China has gradually introduced PM_2.5_ in the national air quality monitoring network and publicized real-time monitoring data since 2013. In this study, the total study period encompassed 2014–2017, and the years of analysis differed by city based on the availability of air pollution data. Of the 184 cities, 78 cities had 3-year PM data records and 106 cities had 4-year PM data records. The years of the study period for each city were presented in **S1 Table**.” (lines 128-142)

10. Results: Line 200: Please provide a range for the number of daily hospital admissions for pneumonia.

*Response*

We thank the Editors for this comment. We have presented the range of daily hospital admissions for pneumonia as “The mean number of daily hospital admissions for pneumonia was 26 (range: 1 to 376).” (lines 254-255)

11. Results: Line 268: Please change “statistically insignificant” to “effects were not significant after controlling for…”

*Response*

Done.

12. Results: Lines 270-277: Please quantify these results with 95% CIs and *P* values, and clarify that the results are presented in S4 Table.

*Response*

Done. (lines 354-368)

13. Discussion: Lines 280-285: Your study is observational and thus causality cannot be inferred. Please revise throughout and remove causal language (such as, “...we assessed the effects of ambient PM pollution on pneumonia admissions…” and “...investigate the acute effects of PM on pneumonia…”).

*Response*

Done. (lines 371-377)

14. Discussion: Lines 289-290: Please revise causal language (“...indicating that short-term PM exposure (for even less than a day) could trigger pneumonia.”)

*Response*

We have rewritten the sentence as “the highest estimates for single-day lag models were observed for lag 0, indicating an immediate association between PM and pneumonia.” (lines 380-382)

15. Discussion: Lines 293-294, Lines 299-300, Line 305,: Please revise causal language.

*Response*

Done.

16. Discussion: Line 307: Please revise the text to avoid over-interpretation of the conclusion here.

*Response*

We thank the Editors for this comment. We have deleted the sentence.

17. Discussion: Line 362-363: Please revise the text to avoid causal language (...”assist in understanding how PM causes lung-inflammation…”)

*Response*

We thank the Editors for this comment. We have deleted the sentence.

18. Figure 1: Please revise Figure 1 such that it is possible to see the locations of the cities more clearly. Please clarify the meaning of the variation in dot size.

*Response*

We thank the Editor for this comment. As requested by Reviewer #1, we have removed all names of cities and only have dots for the cities. The original Figure 1 has two types of dots, and the larger dots indicate the most populous city in each province. We have now drawn the regions by different colors in updated Figure 1.

19. Figure 2: Please provide units for the axes.

*Response*

Done.

20. Table 3 and Table 4, and S2, S3, and S4 tables: Please indicate if these analyses are adjusted for any factors, and if so, please specify which factors are being adjusted for, and provide unadjusted values.

*Response*

We thank the Editors for this comment. The associations between PM and daily hospital admissions for pneumonia are estimated by a two-stage approach. In the first stage, quasi-Poisson regression model is fitted to estimate city-specific associations of hospital admissions for pneumonia associated with PM exposure. In the second stage, a random-effects meta-analysis is conducted to pool the city-specific estimates. In the first stage, several covariates were included in the model, following previous published studies (***N Engl J Med*** 2019;381:705-715; ***JAMA*** 2017;318:2446-2456; ***JAMA*** 2006;295:1127-1134; ***JAMA*** 2008;299:2172-2179; ***BMJ*** 2017;356:j667; ***Am J Respir Crit Care Med*** 2017;196:73-78): 1) a natural cubic spline for time with 7 degrees of freedom (*df*) per year to adjust for seasonality and long-term trends; 2) natural cubic splines for 3-day moving average temperature with 6 *df* and relative humidity with 3 *df* to control for non-linear and delayed effects of meteorology; 3) indicators of day of the week and public holiday. The method and the model used in this study were designed before the analyses were conducted, and the pre-specified statistical analysis plan was present in the Supporting Information (**S1 Appendix**). We have now added the variables that were used for adjustment under each table.

21. Thank you for including the completed STROBE checklist as Supporting Information. When completing the checklist, please use section and paragraph numbers, rather than page numbers. Please add the following statement, or similar, to the Methods: "This study is reported as per the Strengthening the Reporting of Observational Studies in Epidemiology (STROBE) guideline (S1 Checklist)."

*Response*

Done. (lines 167-168)

***Comments from the reviewers:***

***Reviewer #1***

Reviewer #1: I confine my remarks to statistical aspects of this paper. These were very well done indeed and I have no problem recommending publication.

*Response*

We deeply appreciate your careful review of our paper.

***Reviewer #2***

The authors have conducted an interesting analysis on a health outcome that has not been well studied, which could aid in both the overall interpretation of PM effects on respiratory infections generally, but also specifically the potential health implications of PM exposures within China. Generally, the analysis is well conducted, but additional clarification is needed in many instances, and the authors should consider revising the approach used to examine effect modification. The following comments identify those areas and issues for the authors to consider in revising the manuscript:

Throughout the manuscript there is too much reliance on statistical significance in presenting results from the study as well as in the discussion of studies in the introduction and discussion. The authors need to remove this and focus on patterns of associations.

*Response*

We thank the Reviewer for this comment. We have revised the manuscript accordingly. Please see the reply to your detailed comments.

Abstract:

1. Line 28: I would specify that these are hospital admissions because I could see some getting confused as to whether these are hospital admissions or emergency department visits.

*Response*

We thank the Reviewer for this comment. We have rewritten the sentence as “Information on daily hospital admissions for pneumonia for 2014–2017 was collected from the database of Urban Employee Basic Medical Insurance.” (lines 32-33)

2. Line 32: should note here what effect modifiers will be examined, otherwise you don't know which ones until in the results section and seeing Table 5.

*Response*

We thank the Reviewer for this comment. We have rewritten the sentence as “Meta-regression models were used to investigate potential effect modifiers, including cities’ annual-average air pollutants concentrations, temperature, relative humidity, gross domestic product per capita, and coverage rates by the UEBMI.” (lines 36-39)

3. Line 41-42: This does not seem like something that should be in the abstract.

*Response*

We thank the Reviewer for this comment. The journal requires authors to describe the main limitations of the study in the Methods and Findings.

Introduction:

4. Line 69: This does not seem like something that should be in the abstract.

*Response*

We thank the Reviewer for this comment. We have deleted the sentence.

5. Line 72: This doesn't make sense to put here because this paper is focusing on short-term exposure and the estimates here are based on health impact functions for long-term PM_2.5_ exposures. It's not really making the correct point.

*Response*

We thank the Reviewer for this comment. We have deleted the sentence.

6. Line 74: should be association, not correlation.

*Response*

Done.

7. Line 75: From this point on, there needs to be more context put around these studies versus just listing them out and what they found. Something like, some studies have started to report some evidence of an association between air pollution exposure (or PM_2.5_ specifically) and respiratory infections, including pneumonia. After that it will help in discussing these studies and their results. Also, it would be better to not just list studies, but find some commonality amongst them.

*Response*

We thank the Reviewer for this comment. We have revised the paragraph as “Air pollution has emerged as a significant public health problem worldwide (***Lancet*** 2002;360:1233-1242). Despite plausible hypotheses linking air pollution with pneumonia (***Eur Respir J Suppl*** 2003;40:39s-46s; ***Am J Physiol Lung Cell Mol Physiol*** 2003;284:22; ***Environ Health Perspect*** 2002;110(8):749-55; ***Environ Sci Pollut Res Int*** 2016;23:10604-10614), limited evidence of the association is available, especially in adults. Some studies have started to report some evidence of an association between ambient particulate matter (PM) pollution and respiratory infections, including pneumonia. A time-series study suggested an association between fine particulate matter (PM_2.5_, PM ≤2.5 μm in aerodynamic diameter) and increased admissions for respiratory tract infections in Medicare enrollees (aged >65 years) in 204 U.S. urban counties (***JAMA*** 2006;295:1127-1134). Similarly, another study reported significant effects of PM_10_ (PM ≤10 μm in aerodynamic diameter) on pneumonia admissions among 36 U.S. citizens aged 65 years or older (***Am J Epidemiol*** 2006;163:579-588). Strosnider et al. assessed age-specific acute effects of PM_2.5_ on respiratory emergency department visits in 17 U.S. states, and reported that short-term exposure to PM_2.5_ was associated with increased emergency department visits for pneumonia in people aged 19–65 years, but not in people aged <19 or ≥65 years (***Am J Respir Crit Care Med*** 2019;199:882-890). A recent case-crossover study from the U.S. demonstrated significant associations between short-term elevations in PM_2.5_ and greater healthcare utilization for acute lower respiratory infection in both children and adults (***Am J Respir Crit Care Med*** 2018;198:759-766). Host et al. found increased admissions for lower respiratory tract infections following PM_2.5_ exposure in six French cities (***Occup Environ Med*** 2008;65:544-551), and two time-series studies in Hong Kong provided further evidence on the acute effects of PM on respiratory tract infections/pneumonia (***Am J Respir Crit Care Med*** 2013;188:1240-1245; ***Thorax*** 2014;69:1027-1033). However, these studies were conducted primarily in developed countries/cities where characteristics of air pollution and socioeconomic status differ from those in developing countries. The effects of short-term exposure to PM pollution on pneumonia in developing counties require further investigation. China, the largest developing country, has one of the highest PM_2.5_ concentrations worldwide (***Environ Sci Technol*** 2016;50:79-88). However, only several studies to date have explored the associations between PM pollution and respiratory tract infections/pneumonia, and were subjected to single city/hospital study and small sample size (***PLoS One*** 2016;11:e0153099; ***Sci Rep*** 2017;7:8679).” (lines 98-121)

8. Line 87: Throughout it says PM levels, but it should actually be PM concentrations. Need to fix this throughout the paper.

*Response*

Done.

9. Line 88 - 92: This sentence is unclear and needs clarification.

*Response*

We thank the Reviewer for this comment. We have revised the Introduction as “China, the largest developing country, has one of the highest PM_2.5_ concentrations worldwide (***Environ Sci Technol*** 2016;50:79-88). However, only several studies to date have explored the associations between PM pollution and respiratory tract infections/pneumonia, and were subjected to single city/hospital study and small sample size (***PLoS One*** 2016;11:e0153099; ***Sci Rep*** 2017;7:8679). In this study, we assembled the comprehensive and up-to-date morbidity of pneumonia and environmental data in China. We examined the associations between short-term exposures to ambient PM pollution and daily hospital admissions for pneumonia in adults in China between 2014 and 2017.” (lines 119-124).

Methods:

10. Line 98 - 101: This doesn't seem like the correct place for this statement. Also, should make it clearer that if cities did not collect the correct health data they were excluded from the analysis in these sentences.

*Response*

We thank the Reviewer for this comment. We have now clarified the inclusion and exclusion criteria of city in the Method that “Total 184 Chinese cities were finally included in this study. Cities were selected according to the availability of daily hospital admission and air pollution data. Cities with only 1-year hospital admission records were excluded owing to the feasibility of model fit. Individuals’ detailed disease diagnosis (International Classification of Diseases (ICD) code and text of disease diagnosis) was required to identify pneumonia admissions. Cities with no information on disease diagnosis recorded in the database were also excluded. In addition, we applied additional rule to control the quality of the original data. We regressed the city-specific average numbers of pneumonia admission per day on the number of enrollers of the city. Cities with absolute value of standardized residuals > 3 were considered as the outliers, and were removed from the analysis.” (lines 128-137)

11. Line 102 - 104: Should say the years of analysis differed by city. Also, it's not clear what is meant by 3-year data records, health data?

*Response*

We thank the Reviewer for this comment. We have rewritten the sentence as “China has gradually introduced PM_2.5_ in the national air quality monitoring network and publicized real-time monitoring data since 2013. In this study, the total study period encompassed 2014–2017, and the years of analysis differed by city based on the availability of air pollution data. Of the 184 cities, 78 cities had 3-year PM data records and 106 cities had 4-year PM data records. The years of the study period for each city were presented in **S1 Table**.” (lines 137-142)

12. Figure 1: this map is confusing. Unable to clearly distinguish where the cities are located because so many dots. May be clearer to remove all names of provinces and only have dots for the cities. If you still want to detail the most populous cities it can be done in a supplemental table or have the size of the dots change depending on population of the city. Do the size of the dots mean something?

*Response*

We thank the Reviewer for this comment. As requested, we have removed all names of cities and only have dots for the cities. The original Figure 1 has two types of dots, and the larger dots indicate the most populous city in each province. We have now drawn the regions by different colors in updated Figure 1.

13. Study Population:

need to specify the age range examined because in the discussion it says adults, but not defined in the methods section.

*Response*

We thank the Reviewer for this comment. We have specified the age range in the Methods as “The UEBMI covers urban employees and retired employees (aged ≥18 years).” (lines 151-152)

14. Line 121: Should be legal instead of legally.

*Response*

Done.

Statistical analysis:

15. Line 162: what sensitivity analyses? if discussed later should note that.

*Response*

We thank the Reviewer for this comment. As stated in lines 237-244, “We conducted several sensitivity analyses: (1) we tested the potential confounding effects of gaseous pollutants in two-pollutant models; (2) we separately examined the associations of PM with pneumonia in cities with only 3- or 4-year data to compare with the primary analysis; (3) we examined the associations between PM and pneumonia in cities with different levels of population coverage by the UEBMI (<20% and ≥20%); (4) we examined the associations excluding cities with ≤2 monitoring stations; (5) we conducted a sensitivity analysis with additional adjustment of the hospitalization of influenza; (6) model specification was also assessed using alternative *df* for time (6–10 per year) or using penalized spline functions for time and meteorological variables in the model.” We have rewritten the sentence as “This model was in line with those used in previous studies, and was tested in sensitivity analyses described in detail below.” (lines 205-207)

16. Line 168 - 173: This discussion on the concentration-response relationship is unclear and could use a revision. It should be concentration versus exposure response. The last few lines of the paragraph needs additional explanation, it's unclear how 5 coefficients were examined and what the variance-covariance matrix represents. Additional explanation is needed to clarify how statements about the shape of the C-R curve where made.

*Response*

We thank the Reviewer for this comment. We have now provided more details on the concentration-response curve in the Methods as “We plotted the national-average concentration-response curves of the associations of PM with pneumonia admissions using the approach of the Air Pollution and Health—A European Approach (APHEA) project (***N Engl J Med*** 2019;381:705-715; ***Environ Health Perspect*** 2005;113:88-95; ***Am J Respir Crit Care Med*** 2017;196:73-81). Briefly, we replaced the linear term of PM in the main model with a B-spline function with two knots at 60 and 150 μg/m^3^ for PM_2.5_, and 100 and 200 μg/m^3^ for PM_10_, based on the distribution of PM levels in each city. We then estimated five regression coefficients of the spline function and the 5×5 variance-covariance matrix in each city. Finally, we fitted multivariable random-effect models to combine the city-specific components of spline estimates. Variance-covariance matrix is a matrix whose element in the *i*, *j* position is the covariance between the *i*-th and *j*-th elements of a random vector. The regression coefficients derived from the main model with a B-spline function for PM.” (lines 214-220)

17. Line 175: Would be better to say something like: We examined effect modification of the relationship between short-term PM exposure and pneumonia hospital admissions in analyses...

*Response*

We thank the Reviewer for this comment. We have rewritten the sentence as “We examined effect modification of the relationship between short-term PM exposure and pneumonia hospital admissions in analyses by sex, age, and region (northern and southern China).” (lines 222-223)

18. Line 178: The part about annual average PM levels is unclear and needs additional explanation. Actually, the whole discussion on how effect modification was examined should be reconsidered. The approach used in the paper is confusing. It would be easier to interpret results if the authors examined effect modification based on the distribution of the factor examined across cities. For example, like the NMMAPS or APHENA studies where they examined how the risk of mortality changed when moving from the 25th to 75th percentile of the factor examined.

*Response*

We thank the Reviewer for this comment. We have now clarified in the Methods (lines 225-234) as follows: In this study, we fitted meta-regression models to investigate several city characteristics as potential effect modifiers, including cities’ annual-average air pollutants concentrations, air temperature and relative humidity, gross domestic product per capita, and coverage rates by the UEBMI. This approach has been widely used to examine effect modification by city-level characteristics in large multicity studies (***N Engl J Med*** 2019;381:705-715; ***BMJ*** 2018 Oct 31;363:k4306; ***BMJ*** 2017;356:j667; ***Am J Respir Crit Care Med*** 2017;196:73-81; ***PLoS Med*** 2019;16:e1002738). Cities’ annual-average air pollutants concentrations, temperature and relative humidity were calculated from daily measurements during the study period. City-specific relative risk and their confidence intervals (CIs) as the outcome were meta-regressed on each continuous variable of city characteristics. We performed random-effects meta-regression model using city-level estimations from the first stage as: y_i_ = βx_i_ + µ_i_ + e_i_ where y_i_ is the city-specific estimation, x_i_ is the city-level characteristic variable, µ_i_ is the city-specific random effect characterized by the between-city variance, and e_i_ is a normal error term (***Statistics in Medicine*** 22:2693-2710). Random-effects meta-regression can be considered as an extension to random-effects meta-analysis (i.e. DerSimonian and Laird model) that includes study-level (i.e. city-level in our case) covariates. The model is implemented by the metareg function in STATA. The algorithm for random-effects meta-regression firstly estimated the between-study (between-city) variance, and then estimate the coefficients, β, by weighted least squares by using the weights 1/(σ_i_^2^ + τ^2^), where σ_i_^2^ is the standard error of the estimated effect in study (city) i and τ^2^ is the between-study (between-city) variance, advocated by Thompson and Sharp (***Statistics in Medicine*** 1999;18:2693-2708).

Sensitivity Analyses:

19. Line 185 - 186: Clarify, does this mean the authors compared associations between cities with 3 years of data versus 4 years of data?

*Response*

We thank the Reviewer for this comment. We examined the associations of PM with pneumonia in cities with 3-year or 4-year data respectively to compare with the primary analysis. The estimates were presented in **S5 Table**. We did not compare the associations between cities with 3-year data vs. 4-year data.

20. Line 187: Prior to the sentence starting "Fourth" : I would add a sentence prior to these last two analyses to note, that model specification was assessed using these two analyses.

*Response*

Done. (lines 243-244)

21. Line 188: The sentence starting "Fifth", would be beneficial to start the sentence by saying: to examine whether we appropriately specified the regression model used in this analysis, we...

*Response*

Done. (lines 243-244)

Results:

22. Line 216: should clarify whether single day lags or the average of 0-2 day.

*Response*

We thank the Reviewer for this comment. We have now clarified as “City-specific estimates of the associations between 3-day moving average (lag 0–2) concentrations of PM_2.5_ and PM_10_ and hospital admissions for pneumonia are presented in **S2 Table**.” (lines 275-276)

23. Line 219: at the lag days examined instead of at different lag days.

*Response*

Done. (line 278)

24. Line 223: Separate paragraph should start here to talk about effect modification results.

*Response*

Done.

25. Line 224: better to present the risk estimates and confidence intervals than to focus on *P* values.

*Response*

Done. (lines 288-291)

26. Line 228 - 230: I don't recall this being mentioned earlier. How were these delineations defined? Need additional explanation.

*Response*

We thank the Reviewer for this comment. We have now clarified in the Methods as “To explore regional heterogeneity of the associations, we divided the 184 cities into six geographical regions, i.e., East, Middle-south, Southwest, Northwest, North and Northeast, in line with previous studies (***Am J Respir Crit Care Med*** 2017;196:73-81).” (lines 208-209) We have now drawn the regions by different colors in Figure 1.

27. Table 3: this table could be simplified by having the CIs directly next to the percent change. Don't need the *P* value column.

*Response*

Done.

28. Table 4: it's really % increase, not % change, replace PC with % Increase

*Response*

Done.

29. Line 240: based off the figures I wouldn't call the curves linear across the full range of concentrations examined. There is actually some evidence of supralinearity where the curve plateaus at some higher concentration. Granted those concentrations are above 100, but it tends to be consistent with some of the evidence from the Integrated Exposure Response Function. See Burnett et al. (2014) - EHP. This statement "in line with reports from other studies" is not entirely correct because it makes it sound like the other studies also examined pneumonia when there are mortality studies being cited. Could say instead that "this evidence of linearity is consistent with studies examined short-term PM exposure and other outcomes."

*Response*

We thank the Reviewer for this comment. We have rewritten the results as “We noted a slightly nonlinear concentration-response curve between PM_2.5_ and pneumonia admissions where there appear to be a plateau at higher concentrations. This evidence of linearity is consistent with studies examined short-term PM exposure and other outcomes.” (lines 308-310)

30. Line 245: should be concentration-response

*Response*

Done. (line 316)

31. Line 250: From this point forward, it's not clear how this analysis was done. I'm surprised the authors did not examine the distribution across all cities of these variables and then examine how the risk estimate may change when going from the 25th percentile to the 75th percentile of the factor being examined. Additionally, there is no discussion of the influence of annual PM concentrations on the results even though it is in the table.

*Response*

We thank the Reviewer for this comment. We have provided more details on this analysis. Please refer to the reply to the comment #18 above. In brief, we fitted meta-regression models to investigate several city characteristics as potential effect modifiers, including cities’ annual-average air pollutants levels, air temperature and relative humidity, gross domestic product per capita, and coverage rates by the UEBMI. This approach has been widely used to examine effect modification by city-level characteristics in large multicity studies (***N Engl J Med*** 2019;381:705-715; ***BMJ*** 2018 Oct 31;363:k4306; ***BMJ*** 2017;356:j667; ***Am J Respir Crit Care Med*** 2017;196:73-81; ***PLoS Med*** 2019;16:e1002738). Instead of using categories of the factors, city-specific relative risk and their confidence intervals (CIs) as the outcome were meta-regressed on each continuous variable of city characteristics.

We have now presented city-level characteristics in the **S1 Table**.

We have now discussed the influence of annual PM concentrations in the Results as “No evidence was found for effect modification by annual-average PM_2.5_ (percentage increase, -0.019%; 95% CI, -0.113% to 0.075%, *P* = 0.688) or PM_10_ (percentage increase, 0; 95% CI, -0.064% to 0.064%, *P* = 0.997).” (lines 335-337)

32. Line 268 - 269: Again, too much reliance on statistical significance, instead focus on whether the associations remained positive. Please revise to reflect this.

*Response*

We thank the Reviewer for this comment. We have rewritten the results as “The associations of PM_2.5_ and PM_10_ with pneumonia remained after adjusting for SO_2_ (PM_2.5_: percentage increase, 0.22%; 95% CI, 0.07% to 0.37%; PM_10_: percentage increase, 0.12%; 95% CI, 0.02% to 0.22%) and O_3_ (PM_2.5_: percentage increase, 0.31%; 95% CI, 0.16% to 0.46%; PM_10_: percentage increase, 0.20%; 95% CI, 0.09% to 0.30%); the effects remained positive but not significant after controlling for CO (PM_2.5_: percentage increase, 0.12%; 95% CI, -0.03% to 0.27%; PM_10_: percentage increase, 0.06%; 95% CI, -0.04% to 0.16%) and NO_2_ (PM_2.5_: percentage increase, 0.10%; 95% CI, -0.05% to 0.25%; PM_10_: percentage increase, 0.04%; 95% CI, -0.06% to 0.14%).” (lines 346-352)

33. Line 269 - 271: I'm not following this discussion on C-R here. It's not clear. Also, I would not use the word threshold, you've really identified an inflection point where the risk changes due to non-linearities in the C-R curve.

*Response*

We thank the Reviewer for this comment. We have now moved this section up together with the discussion of C-R curve. Please see the lines 312-314 in the revised manuscript.

34. Line 272 - 274: Similar comment as to above, group the model specification analyses together and note whether the results were consistent with the base model.

*Response*

We thank the Reviewer for this comment. We have rearranged the result section for the sensitivity analysis to match with the description in the Methods section. We have rewritten the results for the model specification as “The national-average estimates generated from models with alternative *df* for time (6–10 per year, all *P* < 0.05) or with penalized spline functions for time and meteorological variables (PM_2.5_: percentage increase, 0.31%; 95% CI, 0.17% to 0.45%; *P* < 0.001; PM_10_: percentage increase, 0.22%; 95% CI, 0.12% to 0.32%; *P* < 0.001) were comparable to those generated from the base model ” (lines 364-368)

35. Line 277: clarify minor effects. Slightly attenuated, remained relatively unchanged?

*Response*

We thank the Reviewer for this comment. We have rewritten the results as “the estimates were slightly attenuated after excluding cities with ≤2 monitoring stations (PM_2.5_: percentage increase, 0.30%; 95% CI, 0.15% to 0.46%, *P* < 0.001; PM_10_: percentage increase, 0.21%; 95% CI, 0.10% to 0.32%, *P* < 0.001) or after adjustment of hospitalization of influenza (PM_2.5_: percentage increase, 0.29%; 95% CI, 0.13% to 0.45%, *P* < 0.001; PM_10_: percentage increase, 0.18%; 95% CI, 0.07% to 0.29%, *P* < 0.001).” (lines 360-364)

Discussion:

36. Line 283: need to specify age range examined in the methods section.

*Response*

We thank the Reviewer for this comment. We have specified the age range in the Methods as “The UEBMI covers urban employees and retired employees (aged ≥18 years).” (lines 151-152)

37. Line 284: "acute effects", this is not correct, it's not the acute effects, but to investigate whether short-term PM exposures contribute to increases pneumonia hospital admissions.

*Response*

We thank the Reviewer for this comment. We have rewritten the sentence as “… to investigate whether short-term PM exposures contribute to increases pneumonia hospital admissions on a national scale.” (lines 376-377)

38. Line 287: PM_2.5_ and PM_10_?

*Response*

We thank the Reviewer for this comment. We have rewritten the sentence as “We observed increased risk of pneumonia in association with both PM_2.5_ and PM_10_ at lag 0 and lag 1.” (line 379)

39. Line 288: short-time course? from studies examining short-term PM exposures.

*Response*

We thank the Reviewer for this comment. We have rewritten the sentence as “…in line with previous findings from studies examining short-term PM exposures.” (lines 379-380)

40. Line 289: "for even less than a day", not sure how this is being deduced. We don't actually know this since the exposures being assigned are 24-h avg exposures and not hourly exposures.

*Response*

We thank the Reviewer for this comment. We have deleted the words.

41. Line 291: the evidence examining sub-daily exposures is very limited and this study cannot inform that since the authors are focusing on 24-h avg exposures. An association at lag 0 only indicates an immediate effect, it cannot inform that the effect is due to exposures of a shorter duration because the exposure metric used was 24h average, not individual hour values. Please revise.

*Response*

We thank the Reviewer for this comment. We have deleted the discussions about sub-daily exposures.

42. Line 291 - 292: bringing in sub-daily cardiovascular results does not make sense, and again this study cannot inform sub-daily exposures because it is focusing on 24-avg exposures.

*Response*

We thank the Reviewer for this comment. We have deleted the discussions about sub-daily exposures.

43. Line 292 - 294: a 0-2 lag is still considered an immediate effect. Delayed would be if you say associations at lag 2-5 days. Remove delayed and keep cumulative effect.

*Response*

Done.

44. Line 296: Please see earlier comment on this characterization of the C-R curve.

*Response*

We thank the Reviewer for this comment. We have revised the paragraph as “In this study, we explored the concentration-response curve of the associations between PM and pneumonia hospitalizations. China is one of the most polluted countries worldwide (***Environ Sci Technol*** 2014;48:13834-13843). The average city-specific annual-mean PM_2.5_ and PM_10_ concentrations during the study period were 50 μg/m^3^ and 89 μg/m^3^ respectively, allowing us to assess the concentration-response pattern at high concentrations. The curves plateau at high concentrations, indicating that the risk of pneumonia admission is lower at high concentrations. Similar shape of the association was also observed in other studies (***Am J Respir Crit Care Med*** 2017;196:73-81). The leveling-off at high concentrations might be speculated by that people vulnerable to PM exposure may have developed symptoms and sought treatment before PM concentrations reached high concentrations.” (lines 384-392)

45. Line 297 - 299: this is not the correct interpretation. If a health benefit per ton analysis was occurring sure, this statement could be made, but the analysis is focusing on risk estimates so the proper interpretation is that risk is higher and concentrations lower than some value and they plateau above a value.

*Response*

We thank the Reviewer for this comment. We have revised the paragraph accordingly. Please refer to the response to comment #44 above for details.

46. Line 299 - 300: This sentence is unclear and I don't think it's needed.

*Response*

We thank the Reviewer for this comment. We have deleted the sentence.

47. Line 302 - 304: Jumping to harvesting seems like a stretch. It could just be that PM_2.5_ has a greater health risk than PM_10_ and that PM_10_ is representing exposure to more particles in the coarse range (PM_10-2.5_) than fine particles. I don't think the harvesting statement makes sense based on this study. If the authors examined a longer duration of lags to support this statement then it would be a different story.

*Response*

We thank the Reviewer for this comment. We have revised the paragraph accordingly. Please refer to the response to comment #44 for details.

48. Line 305: But a threshold analysis was not conducted, the analysis only focused on assessing linearity. I don't think this statement is needed. If anything the authors could state the point at which confidence intervals expand and there is less certainty in the shape of the C-R at both lower and higher PM concentrations.

*Response*

We thank the Reviewer for this comment. We have deleted the sentences.

49. Line 307: I'd remove statements about health benefits because this paper is not focusing on that and it is a little confusing.

*Response*

Done.

50. Line 309 - 311: Seems odd to cite a respiratory mortality study instead of a pneumonia HA one here. I would revisit other pneumonia studies to confirm the findings of this study instead of mortality.

*Response*

We thank the Reviewer for this comment. We have deleted the original reference, and cited several pneumonia studies. (lines 394-395)

51. Line 312-313: I think this is a better statement to make about differences between PM_10_ and PM_2.5_ versus the statement above about surface area. The makeup of the particles themselves is much different.

*Response*

We thank the Reviewer for this comment. We have revised the paragraph accordingly “PM_10_ is representing exposure to more particles in the coarse range (PM_10-2.5_) than PM_2.5_. The sources, composition, and lung deposition patterns of PM_10–2.5_ vary from those of PM_2.5_ (***JAMA*** 2008;299:2172-2179; ***Annu Rev Public Health*** 2000;21:309-33; ***J Air Waste Manag Assoc*** 1997;47:1238-1249). The chemical composition of PM differs by size with more crustal materials in PM_10-2.5_ and more combustion-related constituents in PM_2.5_ (***J Air Waste Manag Assoc*** 1997;47:1238-1249). It was reported that PM_2.5_ can penetrate deep into the lungs, reaching the bronchioles and depositing inside the alveoli. In addition, PM_2.5_ has a larger surface area than PM_10_, and thus can absorb more toxic substances per unit mass (***J Air Waste Manag Assoc*** 2006;56:709-742).” (lines 395-401)

52. Line 314: I would delete this sentence, especially since there is no citation.

*Response*

We thank the Reviewer for this comment. We have deleted the sentence.

53. Line 321-322: This paragraph is on the right tract, but it needs more at the end, the PM-metals part really doesn't fit in with the rest of the paragraph.

*Response*

We thank the Reviewer for this comment. We have deleted the PM-metals part and provided more discussion at the end as “…PM-induced oxidative stress might impair the cellular defense and immune system, increasing the susceptibility to infection (***Thorax*** 2014;69:1027-1033). In addition, PM might enhanced the susceptibility to infection through the suppression of the immune response (***J Toxicol Environ Health A*** 1999;57:445-457). These toxicological evidences were consistent with our findings on the association between short-term exposure to PM and pneumonia hospitalizations. However, it should be noted that our study does not reveal the mechanisms related to PM that may be active in these processes, and thus further study is still required to verify the causality.” (lines 408-413)

54. Line 324-325: I couldn't access the supplement. Did they stay positive? Need to better convey the results versus relying on statistical significance.

*Response*

We thank the Reviewer for this comment. We have rewritten the sentence as “In the two-pollutant models, the associations between PM_2.5_ and PM_10_ and pneumonia remained positive but were not significant after adjusting for CO and NO_2_.” (lines 415-416) The detailed estimates have been presented in **S4 Table**.

55. Line 325: From this point on, too much reliance on statistical significance in this whole paragraph.

*Response*

We thank the Reviewer for this comment. We have revised the paragraph accordingly. (lines 416-421)

56. Line 330: Need to specify PM_2.5_.

*Response*

Done. (line 422)

56. Line 352-355: this needs additional explanation. Unclear what the authors are trying to convey.

*Response*

We thank the Reviewer for this comment. In this national time-series study, we applied a two-stage analytic approach to estimate the regional- and national-average associations of PM levels and daily hospital admissions for pneumonia. This approach had been developed and widely applied in previous multicity time-series studies (***N Engl J Med*** 2019;381:705-715; ***JAMA*** 2004;292:2372-2378; ***JAMA*** 2006;295:1127-1134; ***JAMA*** 2008;299:2172-2179; ***BMJ*** 2017;356:j667; ***Am J Respir Crit Care Med*** 2016;194:1233-1241). One-stage method could also be used in such data analysis. Therefore, here we discussed the differences of the two methods. We have expanded accordingly as “both one-stage and two-stage methods could be applied in multicity analysis. In this study, two-stage method was applied considering the issue of the computational complexity (***Int J Epidemiol*** 2010;39:1345-1359), in line with previous studies (references listed above). The variances estimated in the first step in the two-stage analysis may not be exactly those in the one-stage method. However, it was reported that one-stage and two-stage methods often generate very similar results (***Stat Med*** 2000;19;3417-3432).” (lines 452-456)

***Reviewer #3***

This study investigates short term effects of air pollution on risk of pneumonia hospital admissions. This is a well conducted study and well written. Some issues need to be clarified, as outlined below.

1. Line 121: Can the authors remind the reader of the coverage rate of UEBMI?

*Response*

We thank the Reviewer for this comment. We have now presented the coverage rate of UEBMI in the **S1 Table**.

2. Can authors elaborate further on using hospital admissions and not including emergency room visits. Hospital admissions will include the more severe cases only.

*Response*

We thank the Reviewer for this comment. As requested, we have specified that only hospital admissions were included in this analysis. We have also addressed this issue in the Limitations as “In this study, we examined the associations between PM and hospital admissions for pneumonia, not including emergency room visits. As hospital admissions generally include the more severe cases, future investigations are needed to assess the effects of PM on other morbidity outcomes (e.g., emergency room visits).” (lines 456-460)

3. Statistical analysis section: Can authors provide a bit more details on how the degrees of freedom were chosen for the variables that were used in natural cubic splines? I suppose they looked at model fit, but this should be further explained.

*Response*

We thank the Reviewer for this comment. We have provided more details on statistical analysis in the Methods (lines 190-200) as “We used a common two-stage method to examine the associations between PM and pneumonia admissions (***N Engl J Med*** 2019;381:705-715; ***JAMA*** 2008;299:2172-2179; ***BMJ*** 2017;356:j667; ***Am J Respir Crit Care Med*** 2016;194:1233-1241). … In stage 1, we assessed the city-specific effects of PM on pneumonia admissions using a quasi-Poisson regression model that allows for over-dispersed admission counts. Several covariates were included in the model, following previous published studies: 1) a natural cubic spline for time with 7 degrees of freedom (*df*) per year to adjust for seasonality and long-term trends; 2) natural cubic splines for 3-day moving average temperature with 6 *df* and relative humidity with 3 *df* to control for non-linear and delayed effects of meteorology; 3) indicators of day of the week and public holiday. The selection of *df* values was based on the parameter used in several recent large national studies (***N Engl J Med*** 2019;381:705-715; ***Am J Respir Crit Care Med*** 2017;196:73-78; ***Lancet Planet Health*** 2018;2:e12-e18; ***Environ Health Perspect*** 2019;127:17008; ***Environ Health Perspect*** 2017;125:117006; ***Environ Int*** 2018;117:33-39; ***Epidemiology*** 2018;29:482-489).” To test the robustness of the main model, we conducted sensitivity analyses using different *df* values for calendar time (6–10 per year), or using penalized spline functions for time and meteorological variables. The estimates were broadly similar (**S5 Table**).” (lines XX)

4. Line 228: Authors refer to the online supplement when further stratifying regions, but can they provide a description of those findings in the text?

*Response*

We thank the Reviewer for this comment. We have expanded accordingly as “There was a significant heterogeneity in the PM-hospitalization associations across different regions. The associations were more evident in the Middle-south, East, and North regions.” (lines 298-300)

5. Can authors add the variables that were used for adjustment under each table? This is mentioned in the Statistical analysis section, but should also be reported under each table. For example, is Table 5 adjusted for temperature and humidity, in addition to looking at meta-regression models by city-level annual average temperature and humidity?

*Response*

We thank the Reviewer for this comment. We have now added the variables that were used for adjustment under each table. Table 5 presents meta-regression results of the modification effects of city-level characteristics on the associations between PM and hospital admissions for pneumonia. In this study, we fitted meta-regression models to investigate several city characteristics as potential effect modifiers, including cities’ annual-average air pollutants levels, weather conditions, gross domestic product per capita, and coverage rates by the UEBMI. This approach has been widely used to examine effect modification by city characteristics in large multicity studies (***N Engl J Med*** 2019;381:705-715; ***BMJ*** 2018 Oct 31;363:k4306; ***BMJ*** 2017;356:j667; ***Am J Respir Crit Care Med*** 2017;196:73-81; ***PLoS Med*** 2019;16:e1002738). Cities’ annual-average air pollutants levels, temperature and relative humidity were calculated from daily measurements during the study period. City-specific relative risk and their confidence intervals (CIs) as the outcome were meta-regressed on each continuous variable of city characteristics. We performed random-effects meta-regression model using city-level estimations from the first stage as: y_i_ = βx_i_ + µ_i_ + e_i_ where y_i_ is the city-specific estimation, x_i_ is the city-level characteristic variable, µ_i_ is the city-specific random effect characterized by the between-city variance, and e_i_ is a normal error term (***Statistics in Medicine*** 22:2693-2710).

6. Did authors consider looking at multi-pollutant models (i.e. adjusting for more than two pollutants in their models)?

*Response*

We thank the Reviewer for this comment. Previous studies generally fitted single-pollutant model in the main analysis to assess the health effects of air pollution, and further conducted two-pollutant analysis to explore potential confounding by other air pollutants (***N Engl J Med*** 2019;381:705-715; ***Circulation*** 2009;120:949-955; ***BMJ*** 2017;356:j667; ***Am J Respir Crit Care Med*** 2017;196:73-81; ***Am J Respir Crit Care Med*** 2013;188:1240-1245; ***Am J Respir Crit Care Med*** 2016;194:1233-1241). To be consistent with previous studies and also to increase the comparability of the effect estimates across studies, we adopted the same approach in this study.

As requested, we fitted multi-pollutant models including PM_2.5_, PM_10_, SO_2_, NO_2_, CO, and O_3_. A 10-μg/m^3^ increase in 3-day moving average (lag 0–2) concentrations of PM_2.5_ and PM_10_ was associated with a 0.02% (-0.14% to 0.17%, *P* = 0.844) and 0% (-0.11% to 0.10%, *P* = 0.927) increase in hospital admissions for pneumonia, respectively. However, considering the strong correlation between the different air pollutants, we think that this result was too difficult to be well interpreted. Therefore, we didn’t present this in the revised manuscript.

7. Line 276-277: Where are those results reported? Also, why using a cut-off of 20% for health insurance coverage?

*Response*

We thank the Reviewer for this comment. The results of the sensitivity analyses were presented in **S5 Table**. We have also presented detailed estimates in the Results. (lines 354-368)

We selected a cut-off of 20%, because the median value of the coverage rates by the UEBMI across the 184 cities was close to 20% (i.e. 18%). The risk estimates were similar in cities with different levels of population coverage (<20% and ≥20%), which is consistent with our meta-regression results. We fitted meta-regression models to investigate several city characteristics as potential effect modifiers, including the coverage rates by the UEBMI. The coverage rate for health insurance did not significantly modify the associations (*P* > 0.05). Please refer to the reply to the comment #5 for the method we used in the meta-regression to assess the effect modification.

8. Line 298: Can authors provide more discussion on why risks would be higher per unit increase at low levels? This is a very important topic going forward in air pollution epidemiology.

*Response*

We thank the Reviewer for this comment. We have revised the paragraph as “In this study, we explored the concentration-response curve of the associations between PM and pneumonia hospitalizations. China is one of the most polluted countries worldwide (***Environ Sci Technol*** 2014;48:13834-13843). The average city-specific annual-mean PM_2.5_ and PM_10_ concentrations during the study period were 50 μg/m^3^ and 89 μg/m^3^ respectively, allowing us to assess the concentration-response association at high concentrations. The curves plateau at high concentrations, indicating that the risk of pneumonia admission is lower at high concentrations. Similar shape of the association was also observed in other studies (***Am J Respir Crit Care Med*** 2017;196:73-81). The leveling-off at high concentrations might be speculated by that people vulnerable to PM exposure may have developed symptoms and sought treatment before PM concentrations reached high concentrations.” (lines 384-392)

9. Line 316: Possible mechanistic pathways are outlined, but pneumonia is an infection and it seems there are missing discussion points on how air pollution could be increasing the risk of getting this type of infection. Further discussion is needed in this paragraph.

*Response*

We thank the Reviewer for this comment. We have revised the paragraph about the possible mechanistic pathways accordingly. Specifically, about the risk of getting infection, we stated that “PM-induced oxidative stress might impair the cellular defense and immune system, increasing the susceptibility to infection (***Thorax*** 2014;69:1027-1033). In addition, PM might enhanced the susceptibility to infection through the suppression of the immune response (***J Toxicol Environ Health A*** 1999;57:445-457). These toxicological evidences were consistent with our findings on the association between short-term exposure to PM and pneumonia hospitalizations. However, it should be noted that our study does not reveal the mechanisms related to PM that may be active in these processes, and thus further study is still required to verify the causality.” (lines 408-413)

10. Can authors describe why they did not adjust for influenza epidemics in their models? This is a common approach when looking at short term air pollution-respiratory disease associations.

*Response*

We thank the Reviewer for this comment. In the regression model, a natural cubic spline function of calendar time with seven degrees of freedom (*df*) per year was included to adjust for seasonality and for other time-varying influences on admissions (eg, influenza epidemics and longer-term trends due to changes in medical practice patterns). As a result, little information at the time scales of longer than 2 months would be retained in estimating the risks, considering that the time-series analysis was in fact a self-control design where participants were compared with themselves at different levels of air pollutants. Therefore, the impact of influenza epidemics is expected to be minor. This might explain why influenza epidemics was not considered in many previous large multicity time-series studies (***N Engl J Med*** 2019;381:705-715; ***JAMA*** 2004;292:2372-2378; ***JAMA*** 2006;295:1127-1134; ***JAMA*** 2008;299:2172-2179; ***BMJ*** 2017;356:j667; ***Am J Respir Crit Care Med*** 2017;196:73-81).

As requested, we conducted a sensitivity analysis with additional adjustment of hospitalization of influenza. The estimates changed little. A 10-μg/m^3^ increase in 3-day moving average (lag 0–2) concentrations of PM_2.5_ and PM_10_ was associated with a 0.29% (0.13 to 0.45) and 0.18% (0.07% to 0.29%) increase in hospital admissions for pneumonia, respectively. The results were presented in the **S5 Table.**

11. Concentration-response curves: Can authors add the 24-hour standards for PM_10_ and PM_2.5_ as vertical dashed lines in the graphs? This would give a sense of what standards are currently and how effects are being observed in regards to those standards.

*Response*

Done.

***Reviewer #4***

The manuscript assesses the relationship between short-term exposure to particulate matter (PM) and hospital admissions for pneumonia in China for a 4-years period (2014-2017). The key finding is that PM is associated with increased pneumonia admissions, especially in the elderly population. Moreover, effects are stronger in cities with higher temperatures and relative humidity.

The study bases on a huge dataset with data from 184 cities across China, representing differing climates across the country. Further, the question and the results are of interest to general practitioners and pulmonary specialists as well as public health officials; the topic is quite timely as air pollution is still affecting populations worldwide, even at places with low air pollution levels. The manuscript is well written and the statistical approach is appropriate. However, I have some concerns:

**Major comments:**

1. My biggest concern is about the selection of pneumonia as the only outcome. For example, it would be interesting to look also at respiratory infections or influenza. Further, individuals with certain chronic medical conditions, such as COPD - which includes emphysema and chronic bronchitis - and asthma are especially at risk for pneumonia. Therefore, I strongly suggest including further outcomes.

*Response*

We thank the Reviewer for this comment. We agree with you that other respiratory outcomes may be also of interest. However, the primary objective of this paper is to focus on the effect of PM on pneumonia. Clinically, pneumonia, influenza, COPD and asthma are different entities, though they all belong to respiratory disease. Moreover, in our study, hospital admissions for each health condition were identified based on the principle diagnoses. Both ICD-10 code and natural language processing (text of disease diagnosis) were used. Natural language processing has been validated to be an efficient method for identifying cases in large clinical databases (***J Am Med Inform Assoc*** 2011;18:544-551). Using both ICD-10 code and natural language processing could significantly reduce the impact of coding inaccuracy (***Stroke*** 2017;48:2052-2059). Consequently, extraction of a new diagnosis, e.g. COPD or asthma, requires substantial work before the analysis can be carried out. Therefore, data related to COPD or asthma is not available at the moment. As requested by several reviewers, with certain preliminary related work done before submission, we have tried to extract the hospitalization of influenza in our database in this revision. However, considering the nature of influenza where much more cases may be only mild without the need of hospitalization and the fact that the diagnosis of influenza requires the etiological examination, we think the hospitalization of influenza in our database can only be a rough approximation without the verification of the national surveillance of influenza from the National Center of Disease Control. Therefore, given the main aim of the paper, we only use this information for the sensitivity analysis, i.e. assessment of the association between PM and pneumonia with additional adjustment on the hospitalization of influenza (Please see the reply to the comment below).

2. Statistical analysis, lines 152-161: As the association between PM and pneumonia might be confounded by influenza, I suggest adjusting for influenza in a sensitivity analysis.

*Response*

We thank the Reviewer for this comment. As requested, we conducted a sensitivity analysis adjusting for the hospitalization of influenza. The estimates changed little. A 10-μg/m^3^ increase in 3-day moving average (lag 0–2) concentrations of PM_2.5_ and PM_10_ was associated with a 0.29% (0.13 to 0.45) and 0.18% (0.07% to 0.29%) increase in hospital admissions for pneumonia, respectively. The results were presented in the **S5 Table.**

3. Statistical analysis, lines 163-166: Why did the authors choose lags of 0, 1 and 2 days? Previous studies have seen more delayed effects of PM, especially with respiratory diseases.

*Response*

We thank the Reviewer for this comment. In this study, the associations between PM and hospital admissions pneumonia were not significant at longer lag days, in line with previous findings (***Am J Epidemiol*** 2006;163:579-588; ***Am J Respir Crit Care Med*** 2013;188:1240-1245; ***Am J Respir Crit Care Med*** 2017;196:73-81). Therefore, only lags of 0, 1 and 2 days were presented in this study.

4. Discussion, lines 324-335: The authors could also assess effect modification by other pollutants (NO_2_ or CO) using categories for pollutant levels.

*Response*

We thank the Reviewer for this comment. As requested, we have assessed effect modification by NO_2_ or CO. NO_2_ or CO did not significantly modify the associations between PM and hospital admissions for pneumonia (*P* > 0.05). The results have been presented in the updated **Table 5**.

5. Discussion, lines 337-343: The authors need to provide a more detailed discussion and potential mechanisms for their finding of stronger effects in cities with higher temperature and relative humidity.

*Response*

We thank the Reviewer for this comment. As requested, we have elaborated the discussion as “The effects were stronger in cities with higher temperatures (or higher relative humidity since they are highly correlated). There are several possible explanations. High temperature itself is associated with increased risk of pneumonia (***Epidemiology*** 2012;23:813-820; ***Thorax*** 2009;64:507-511). It was reported that high temperature impacts the emission, transportation, dilution, chemical transformation, and deposition of pollutants (***Sci Total Environ*** 2005;342:5-86). Thus there may exist the interaction between high temperatures and PM. Previous studies also show moderate evidence of the modifying effect of temperature on PM_10_. A meta-analysis reported that the relative risks for respiratory mortality per 10 μg/m^3^ increase in PM_10_ in the low, middle, and high temperature level were 1.005 (1.000 to 1.010), 1.008 (1.006 to 1.010), and 1.019 (1.010 to 1.028), respectively (***Environ Pollut*** 2017;224:326-335). This is consistent with our findings. In addition, the larger estimate might be associated with exposure patterns. People generally spend more time outdoors in warmer cities, resulting in smaller measurement errors (***Sci Total Environ*** 2013;451:259-265).” (lines 431-441)

**Minor comments:**

1.	Abstract, line 31: The authors should drop "overdispersed" here as the use of a quasi-Poisson model already implies consideration of overdispersion. Further, the authors should add here that they adjusted for confounding.

*Response*

We thank the Reviewer for this comment. We have rewritten the sentence as “Associations of PM concentrations and hospital admissions for pneumonia were estimated for each city using a quasi-Poisson regression model controlling for time trend, temperature, relative humidity, day of the week and public holiday, and then pooled by random-effects meta-analysis.” (lines 34-36)

2. Study sites, lines 96-104: The authors should give more details on the choice/inclusion of cities. Why, for example, is Beijing not included, although it is the capital of China? What about Shanghai? What was the original number of cities? Further, authors should provide more details on what they mean with "Cities with no information on ICD code".

*Response*

We thank the Reviewer for this comment. We have provided more details on the choice/inclusion of cities in the Methods that “Total 184 Chinese cities were finally included in this study. Cities were selected according to the availability of daily hospital admission and air pollution data. Cities with only 1-year hospital admission records were excluded owing to the feasibility of model fit. Individuals’ detailed disease diagnosis (International Classification of Diseases (ICD) code and text of disease diagnosis) was required to identify pneumonia admissions. Cities with no information on disease diagnosis recorded in the database were also excluded. In addition, we applied additional rule to control the quality of the original data. We regressed the city-specific average numbers of pneumonia admission per day on the number of enrollers of the city. Cities with absolute value of standardized residuals > 3 were considered as the outliers, and were removed from the analysis. China has gradually introduced PM_2.5_ in the national air quality monitoring network and publicized real-time monitoring data since 2013. In this study, the total study period encompassed 2014–2017, and the years of analysis differed by city based on the availability of air pollution data. Of the 184 cities, 78 cities had 3-year PM data records and 106 cities had 4-year PM data records. The years of the study period for each city were presented in **S1 Table**.” (lines 128-142) The original number of cities is 218. In this study, Beijing and Shanghai were not included because of the lack of the detailed diagnosis of disease in the national database.

3. Study population: As the study uses data from the UEBMI system, results are not transferable to the rural population. This should be acknowledged in the limitations.

*Response*

We thank the Reviewer for this comment. We have now addressed this issue in the Limitations as “Only urban employed and retired individuals were included in this study based on the type of insurance enrolled. Due to the differences in sociodemographic characteristics and PM levels between rural and urban areas, the generalizability of our findings should be interpreted with caution.” (lines 449-452)

4. Lines 124-125: Please provide an ICD code here.

*Response*

Done. (lines 161-162)

5. Environmental data: The authors state that they obtained hourly air pollution concentrations. How did they calculate 24-hour averages? Was the exclusion of "days with missing monitoring measurements" done for cities with only one monitoring site?

*Response*

We thank the Reviewer for this comment. We calculated 24-hour averages by averaging hourly air pollution measurements within a day. As the rates of missing data were only 0.66% for PM_2.5_ and 0.15% for PM_10_, we excluded days with missing monitoring measurements from the analysis. The exclusion of "days with missing monitoring measurements" was done for cities with only one monitoring site.

6. Lines 178-179: Please be more specific here and replace "weather conditions" with "air temperature and relative humidity".

*Response*

Done. (line 227)

7. Results: The authors need to give more details on the cities included. For example, what is the population of the cities? What is the UEBMI coverage per city? What is the distribution of daily hospital admissions, air pollutants and meteorological variables.

*Response*

We thank the Reviewer for this comment. We have now presented city-specific population, the number of individuals enrolled in the database, the UEBMI coverage rate, daily pneumonia admissions, air pollutants levels, and meteorological variables in the **S1 Table.**

8. Figure 2: Please provide units for the axes.

*Response*

Done.

9. Table 5: Please mention the increment for the effects estimates.

*Response*

We thank the Reviewer for this comment. The increment for the effects estimates have now been presented in the parentheses in **Table 5**.

10. Line 272: Why did the authors choose 100 µg/m³ as cut-off? The decline in the exposure-response function starts earlier.

*Response*

We thank the Reviewer for this comment. We plotted the national-average concentration-response curves of the associations of PM with pneumonia admissions using the approach of the Air Pollution and Health—A European Approach (APHEA) project (***N Engl J Med*** 2019;381:705-715; ***Environ Health Perspect*** 2005;113:88-95; ***Am J Respir Crit Care Med*** 2017;196:73-81). Briefly, we replaced the linear term of PM in the main model with a B-spline function with two knots at 100 and 200 μg/m^3^ for PM_10_, based on the distribution of PM levels in each city. Therefore, following visual inspection of the concentration-response curve as well as considering the knot position of the spline function, we decided to use the cut-off of 100 µg/m³.
