# Supplementary material for: Ambient particulate matter pollution and adult hospital admissions for pneumonia in urban China: A national time series analysis for 2014 through 2017
Source: PLoS Med. 2019 Dec 31;16(12):e1003010. doi: 10.1371/journal.pmed.1003010 (PMC6938337; doi:10.1371/journal.pmed.1003010)
Supplement: Attachment — Submitted filename: Response to Editors and Reviewers.docx [file pmed.1003010.s009.docx]

**Responses to comments of editors and reviewers**

***EDITORIAL:***

1. Introduction: Lines 118-119: Please remove the sentence: “In this study, we assembled…” as the meaning is not clear.

*Response*

Done.

2. Results: Line 351: In the sentence “We observed the same trend…” the term "trend" is used to refer to a nonsignificant P value. The term trend should be used only when the test for trend has been conducted. Please revise to: “We observed the same direction of effect modification by temperature (P = 0.129) and relative humidity (P = 0.378) on the association between PM_2.5_ and pneumonia, but the effects were not statistically significant.”

*Response*

Done.

3. Table 2: Please provide p values associated with all statistical comparisons, and please include the specific value for each comparison, unless p<0.001 (as you have already noted). Please also include the specific p values where p is greater than 0.05.

*Response*

Done.

4. Table 3, Table 4, S2 Table, S3 Table, S4 Table, and S5 Table: You have noted that results presented in these tables reflect analyses adjusted for covariates including temperature, humidity, calendar time, day of the week and public holiday. Thank you for your note to the editors regarding the unadjusted analyses. However, we ask that you also present the results of the unadjusted analyses in the tables.

*Response*

Done.

5. S2 Table, S3 Table, S4 Table, and S5 Table: Please define the abbreviation for “PM”.

*Response*

Done.

***FIGURES and GRAPHICS:***

1. Please include the location from which you obtained the base map used in [specific figure] in the legends for this figure. Please note that, because all PLOS articles are published under a CC BY license (creativecommons.org/licenses/by/4.0/), we cannot publish proprietary maps such as Google Maps, Mapquest or other copyrighted maps. If your map was obtained from a copyrighted source please amend the figure so that the base map used is from an openly available source. Unfortunately, it is unlikely that a source from the Ministry of Natural Resources of China would produce a base map that we are able to publish under our copyright.

The following websites are good examples of where you can source open access or public domain maps:

* World of Maps - Many, but not all, of the maps are in the public domain so please check the usage rights before downloading. (http://www.worldofmaps.net)

* U.S. Geological Survey (USGS) - All maps are in the public domain. (http://www.usgs.gov)

* PlaniGlobe - All maps are published under a Creative Commons license so please cite “PlaniGlobe, http://www.planiglobe.com, CC BY 2.0” in the image credit after the caption. (http://www.planiglobe.com/?lang=enl)

* Natural Earth - All maps are public domain. (http://www.naturalearthdata.com/about/terms-of-use/)

*Response*

Done.

***METADATA:***

1. Please ensure that the funders and grant numbers match between the Financial Disclosure field and the Funding Information tab in your submission form. Note that the funders must be provided in the same order in both places as well. Currently, your funders are not listed in the same order in both places.

*Response*

Done.
